# SWAET 2018: Scandinavian Workshop on Applied Eye Tracking Abstracts 23‐24 August, 2018 Copenhagen Business School Solbjerg Plads 3, SP2.01 2000 Frederiksberg DENMARK Edited by Daniel Barratt, Raymond Bertram, & Marcus Nyström

**DOI:** 10.16910/jemr.11.5.1

**Published:** 2018-12-21

## Table of contents

**KEYNOTE LECTURES **

**EYE TRACKER PRESENTATIONS **

**ORAL PRESENTATIONS**

**SESSION 1: Eye-tracking technology: Latest developments **

**SESSION 2: Language processing **

**SESSION 3: Special populations **

**SESSION 4: Eye-tracking measures: Fixations, saccades, microsaccades, and pupil size **

**SESSION 5: Reading comprehension in children and adults **

**SESSION 6: Consumer and gaming behaviour **

**SESSION 7: Social and temporal illusions **

**SESSION 8: Film subtitles and musical notation **

**POSTER PRESENTATIONS**

**Eye-tracking methodology **

**Language processing and reading comprehension **

**Visual search and scene perception **

**Consumer behaviour and logical reasoning **

## KEYNOTE LECTURES

### Relations between fixation locations and fixation durations during reading

Reinhold Kliegl, Professor, Ph.D.

Division of Cognitive Psychology, University of Potsdam, Germany

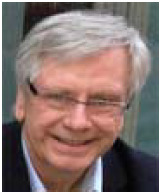



Reinhold Kliegl is professor of experimental psychology at the University of Potsdam, Germany. His research focuses on how the dynamics of language-related, perceptual, and oculomotor processes subserve attentional control, using reading, spatial attention, and working memory tasks as experimental venues. He also examines neural correlates and age-related differences in these processes. His research has been carried out in interdisciplinary projects with colleagues from linguistics as well as from theoretical physics and mathematics.

### Exploring scene context and its effect on eye movement guidance

Monica Castelhano, Associate Professor, Ph.D.

Department of Psychology, Queen’s University, Ontario, Canada

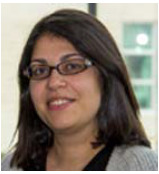



Monica Castelhano is an associate professor in psychology at Queen’s University, Ontario, Canada. Her primary research interests are visual attention and visual memory, and how they function in our everyday lives:

Of all the tasks we perform every day, there is one task that we engage in repeatedly and often without awareness: visual search. Whether looking for your car keys, wallet or simply where your mug is to have a sip of coffee, we engage in this simple task before almost every action. Visual information presented to us at any given moment from the real-world is complex and ever-changing. Consequently, one of the most surprising feats of our cognitive system is the ease with which we can perceive, identify and act upon the world around us. In my lecture, I will explore the various ways that scene context influences and guides eye movements. Rather than a singular influence, we’ll unpack what is meant by scene context and examine the distinction between spatial, semantic, object-scene relations, and object function. Taken together, they improve our understanding of how we process complex information in the real-word and how we are able to perform such complex visual search tasks with relative ease.

## EYE TRACKER PRESENTATIONS

### Presentation of Tobii eye trackers

Nina Chrobot

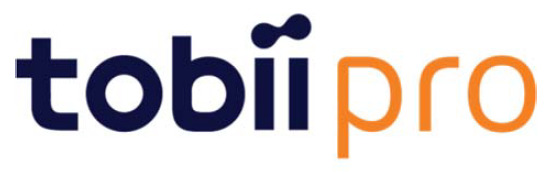



### Presentation of SR Research eye trackers

Sam Hutton

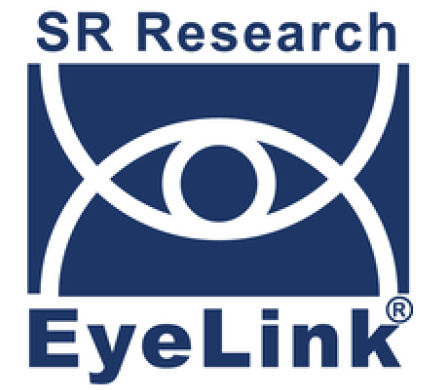



### Presentation of iMotions

Kerstin Wolf

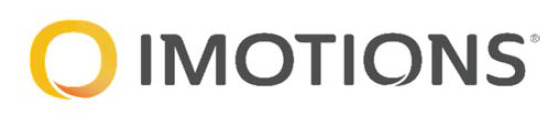



## SESSION 1: Eye-tracking technology: Latest developments

### Data quality in eye trackers: Signal resolution

Kenneth Holmqvist¹, Diederick C. Niehorster², & Pieter Blignaut³

¹ Regensburg University, Germany

² Lund University, Sweden

³ University of Free State, South Africa

For evaluating whether the data from an eye tracker are precise enough for measuring microsaccades, Poletti and Rucci (2016) advocate that the measure “resolution” be used rather than the more established RMS-S2S. Resolution needs to be measured using an artificial eye that can be turned in very small steps, and visual estimation is used to assess whether the movements are visible in the recorded data from the eye tracker. As such, resolution cannot be measured with human data. Currently, resolution has an unclear and entirely uninvestigated relationship to existing RMS-S2S and STD measures of precision (Holmqvist & Andersson, 2017, p. 190). Resolution measurements have only been made on the DPI and one other eye tracker. We do not know resolution values for the most used eye trackers.

In this talk, we present a mechanism – the Stepperbox – for moving artificial eyes arbitrary distances from 1 arcmin and upward. We first present a validation of the mechanism that shows that it is capable of reliably making these movements.

We then use the Stepperbox to find the smallest reliably detectable movement in multiple eye trackers and empirically investigate how resolution relates to the extent (STD) and velocity (RMS-S2S) of noise produced by these eye trackers. Figure 1 shows one of our recordings.

**Figure. 1:**
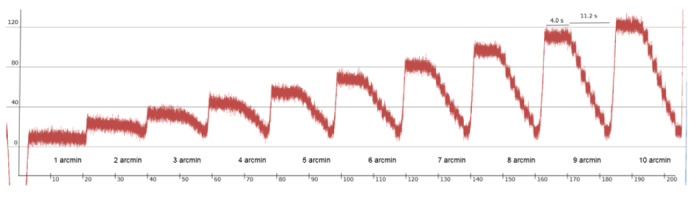
Increasingly larger steps from 1 arcmin to 10 arcmin steps. Each staircase shape involved 10 movements of identical amplitude after a 4 s waiting period. Stops between steps are 1 s long. The smaller movements clearly drown in the noise of this Tobii TX300 eye tracker, and resolution is 6-7 arcmin.

A preliminary analysis indicates that the RMS-S2S values have a *linear relationship* to the resolution values. Eye trackers with filters (coloured noise) differ slightly from eye trackers with no filtering (white noise). We take our results to show that RMS-S2S can be used to assess the minimal amplitude movement that can be reliably detected with an eye tracker. We argue that Poletti and Ruccis’ criticism of RMS-S2S hides a conceptual confusion of resolution as the amplitude where events begin to drown in noise vs. resolution as quantization of the measurement space.

**References**

- Holmqvist, K., & Andersson, R. (2017). *Eye tracking: A comprehensive guide to methods, paradigms, and measures*. Lund, Sweden: Lund Eye-Tracking Research Institute.
- Poletti, M., & Rucci, M. (2016). A compact field guide to the study of microsaccades: Challenges and functions. *Vision Research*, 118, 83-97. doi: 10.1016/j.visres.2015.01.018

### Implicit eye-tracker calibration for seamless interactive setups

Iyad Aldaqre

SR LABS Srl, Milan, Italy

Eye-tracking technology has been used in numerous fields, spanning from basic research, in which gaze data is recorded for later analysis, to interactive situations, in which the direction of gaze is used to control a computer program (Duchowski, 2007). However, for an eye tracker to work properly, it is necessary for the user to go through a calibration phase before being presented with the actual stimulus or interactive event (Poole & Ball, 2006). In a real-life situation, this can limit the experience and deter potential users who might find the procedure complicated and time consuming.

To overcome this limitation, we developed an implicit calibration system that allows instant access to accurate gaze data without the need for a traditional calibration phase. By continuously analysing scene images to estimate areas with higher saliency, the system dynamically learns the best parameters of a polynomial model fit that adjusts gaze data from an uncalibrated eye tracker, maximizing the probability of bringing gaze points where the user is expected to be looking. This procedure leads to an improved accuracy with longer exposure times to the stimulus.

Results show up to 67% improvement in accuracy and 12% in precision of gaze data using the current calibration system compared to an uncalibrated eye tracker (all *p*s < 0.007). Moreover, no differences were found between the adaptive calibration and the manufacturer’s standard calibration either in accuracy or in precision (all *p*s > 0.3). These results show that, when scene information is available to train the model, it can be used instead of a standard calibration without compromising either accuracy or precision.

The current system opens the possibility for spontaneous interactive setups using eye-tracking technology. Unlike other systems (e.g., Pfeuffer et al., 2013; Zhu & Ji, 2005), it eliminates the traditional calibration phase completely and it can be used with commercially available eye trackers. To achieve higher accuracy, more information about the scene can be integrated, like user behaviour, sensor data, and semantic segmentation of the scene. It is also possible to use the system with real objects, like inside an airplane cockpit or an automobile dashboard. Such situations are still to be tested.

**References**

- Duchowski, A. (2007). *Eye tracking methodology: Theory and practice* (2nd ed.). London, UK: Springer-Verlag.
- Pfeuffer, K., Vidal, M., Turner, J., Bulling, A., & Gellersen, H. (2013). Pursuit calibration: Making gaze calibration less tedious and more flexible. In *Proceedings of the 26th annual ACM symposium on user interface software and technology* (pp. 261-270).
- Poole, A., & Ball, L. J. (2005). Eye tracking in human-computer interaction and usability research: Current status and future prospects. In G. Claude (Ed.), *Encyclopedia of human computer interaction*. Pennsylvania, PA: Idea Group Inc.
- Zhu, Z., & Ji, Q. (2005). Eye gaze tracking under natural head movements. In *2005 IEEE Computer Society Conference on Computer Vision and Pattern Recognition – CVPR’05* (pp. 918-923). doi: 10.1109/CVPR.2005.148

### Eye movement labelling in head-mounted display experiments

Ioannis Agtzidis & Michael Dorr

Technical University of Munich, Germany

The segmentation of a gaze trace into the respective eye movement types has been at the centre of eye-tracking research since the first eye trackers appeared. So far, the predominant stimuli in eye-tracking experiments have been static images in which only fixations and saccades are present. In recent years, many researchers have moved to motion pictures and more specifically to dynamic natural scenes in order to approximate more naturalistic viewing behaviours. Notably, this also introduces smooth pursuit (SP) eye movements. Head-mounted displays (HMDs) with eye tracking, which have recently become more popular, are promising tools to get even closer to natural viewing behaviour because they allow free head movements.

There is a plethora of algorithms that detect fixations and saccades, and plenty of data sets to evaluate their performance for both static and dynamic stimuli. For SP detection, substantially fewer algorithms exist and only very few ground-truth data sets for their validation are available. As we move to HMD experiments, more types of eye movements appear such as vestibulo-ocular reflex and optokinetic nystagmus, and the standard algorithms for eye movement detection are not directly applicable. Thus, the creation of ground-truth data sets for 360-degree videos can also be very challenging. Our contribution is two-pronged: (1) a format to represent eye-tracking data in 360- degree equirectangular videos; (2) an open-source hand-labelling tool that allows the hand-labelling of eye within head gaze coordinates and head+eye coordinates.

For the representation of the experimental data, we used the ARFF data format modified for eye-tracking experiments as described in (Agtzidis, Startsev, & Dorr, 2016). Furthermore, we added the headset field of view in degrees and pixels as metadata information. As attributes, the file contains the X and Y equirectangular eye and head coordinates plus the head tilt in degrees. By having the default and the extra two metadata along with these five attributes, we can represent the data in polar coordinates since head translation is not taken into account in simple 360-degree videos.

The open-source hand-labelling tool is an extension of the tool presented in (Agtzidis, Startsev, & Dorr, 2016). Apart from the previous functionality, the new version of the tool can process the extended ARFF format as above. Additionally, it can exactly display gaze traces within the field of view that was visible during the experiment, and thus enables the dissociation of head and eye movements. An example of the tool is presented in Figure 1.

**Figure. 1:**
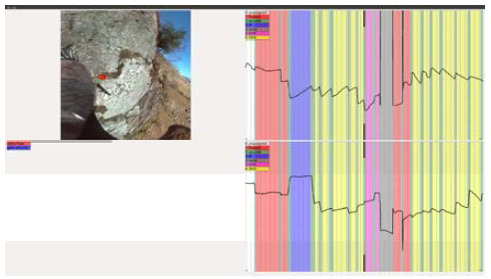
Screenshot of the hand‐labelling tool with field of view presentation enabled and fully annotated eye movements.

Our new tool opens new lines of analysis of ecologically more valid, unconstrained viewing behaviour. The geometric transforms used in this tool may also prove useful for future adaptations of existing algorithms to 360-degree equirectangular videos.

**References**

- Agtzidis, I., Startsev, M., & Dorr, M. (2016). In the pursuit of (ground) truth: A hand-labelling tool for eye movements recorded during dynamic scene viewing. In *2016 IEEE Second Workshop on Eye Tracking and Visualization – ETVIS*. doi: 10.1109/ETVIS.2016.7851169

### Tobii or not Tobii? Assessing the validity of eye-tracking data: Challenges and solutions

Coralie Vincent¹, Efstathia Soroli², Helen Engemann³, Henriëtte Hendriks⁴, & Maya Hickmann¹

¹ CNRS & University of Paris 8, France

² CNRS & University of Lille, France

³ University of Mannheim, Germany

⁴ University of Cambridge, UK

Eye tracking (ET) methods become more and more popular in psycholinguistic research because they offer the possibility to record visual processing in real time, allowing for the study of the relation between cognition and language, two systems often considered independent (Pinker, 1994).

In order to evaluate the impact of specific language properties on online visual processing, we coupled a production task with an ET paradigm. A total of 473 native speakers of two typologically different languages (234 English and 239 French) within three age groups (142 seven-year-old children, 155 ten-year-old children, and 176 adults) were tested in a production task involving 36 dynamic motion scenes (videos), that first had to be visually explored and then verbally described (e.g., “a man running up a hill”).

With respect to the ET data, which is the main focus of the present paper, and in order to properly compare the gaze patterns of the groups, a thorough validity check (pre-processing and quality assessment) was necessary. Indeed, validity is an issue that is almost never addressed in psycholinguistic research, even though an increasing number of researchers report it as one of the main sources of methodological bias (Holmqvist et al., 2011). Apart from the fact that a recording may include segments that are irrelevant for the analysis (e.g., eye blinks, off-screen fixations), it has been found that low quality data may misleadingly point to group differences in gaze behaviour, for instance between adults and children (Wass et al., 2014). More specifically, low precision due to incorrect gaze detection may “flatten out” the gaze distribution across different areas of interest (AOIs) or across groups, while low robustness (i.e., resulting from missing or fragmented data) can make *visit durations* seem shorter than they actually are, and thus bias interpretation of results.

The present paper compares results obtained with a turnkey solution (namely, Tobii Studio) to results obtained with in-house developed algorithms that: (a) carefully discard irrelevant parts of the recording; (b) exclude gaze initiation latencies; and (c) detect and compensate for spatial inaccuracies of the ET data. The findings show that turnkey solutions may be only relevant for some designs (i.e., more appropriate for static/picture material). However, design-adapted validity checks (pre-processing the recordings and quality assessment) as well as target-related compensations of inaccuracies, as proposed in this paper, are crucial and should be common practice for researchers who wish to compare gaze patterns or to evaluate group differences objectively. Challenges related to typical ET measures, such as *gaze proportions* to different dynamic AOI and *visit durations* are also discussed as they seem to be sensitive and subject to change due to validity-related factors.

**References**

- Holmqvist, K., Nyström, M., Andersson, R., Dewhurst, R., Jarodzka, H., & Weijer, J. van de. (2011). *Eye tracking: A comprehensive guide to methods and measures* (1st ed.). New York, NY: Oxford University Press.
- Pinker, S. (1994). *The language instinct: How the mind creates language*. New York, NY: William Morrow & Company.
- Wass, S. V., Forssman, L., & Leppänen, J. (2014). Robustness and precision: How data quality may influence key dependent variables in infant eye‐tracker analyses. *Infancy*, 19(5), 427‑460.

### Is the Tobii Pro Spectrum a useful tool for microsaccade researchers?

Marcus Nyström^1^, Diederick C. Niehorster^1,2^, Richard Andersson^3^, & Ignace T. C. Hooge^4^

^1^ Humanities Lab, Lund University, Sweden

^2^ Department of Psychology, Lund University, Sweden

^3^ Tobii AB, Stockholm, Sweden

^4^ Experimental Psychology, Helmholtz Institute, Utrecht University, Netherlands

Throughout the history of eye movement research, the exact properties of microsaccades have been debated (Collewijn & Kowler, 2008). Part of the reason is differences in instrumentation (Nyström et al., 2016). Therefore, the introduction of a new eye tracker to record fixational eye movements should always be followed by careful investigation of its data quality and a comparison against currently used and established tools.

We recorded eye movements from four people with a newly introduced stereo camera eye tracker (Tobii Pro Spectrum, 600 Hz and 1200 Hz) and the standard eye tracker in the field (EyeLink 1000 Plus, filtered and unfiltered) during a fixation task. Microsaccades were clearly visible in both systems, and comparable microsaccade rates and amplitudes were found when applying a standard algorithm for microsaccade detection (Engbert & Kliegl, 2003). Precision, defined as the root mean square (RMS) of intersample distances, was similar across the systems in the horizontal direction. However, vertical RMS was a factor two lower in the data recorded with the EyeLink compared with the Tobii Pro Spectrum, indicating higher precision.

We conclude that the Tobii Pro Spectrum is a useful tool for microsaccade researchers.

**References**

- Collewijn, H., & Kowler, E. (2008). The significance of microsaccades for vision and oculomotor control. *Journal of Vision*, 8(14), 20.1-2021. doi: 10.1167/8.14.20
- Engbert, R., & Kliegl, R. (2003). Microsaccades uncover the orientation of covert attention. *Vision Research*, 43(9), 1035-1045.
- Nyström, M., Hansen, D. W., Andersson, R., & Hooge, I. (2016). Why have microsaccades become larger? Investigating eye deformations and detection algorithms. *Vision Research*, 118, 17-24. doi: 10.1016/j.visres.2014.11.007

## SESSION 2: Language processing

### Eye movements as a window onto construal in language

Srdan Medimorec, Petar Milin, & Dagmar Divjak

University of Sheffield, UK

Languages provide different ways to express real-world situations (e.g., their properties, actors, and the relations among them). For example, speakers can choose between using the active or passive voice to express the same situation. Cognitive linguists use the theoretical concept of “construal” (Langacker, 1987) to account for these alternating ways of expression. Even though construal has the potential to provide insight into the relation between conceptualization and language choice, there is a dearth of empirical research investigating construal phenomena. Thus far, most attempts to explain linguistic choices by appealing to alternative construals have relied heavily on the analysts’ own intuitions about the data. In the current study, we set out to investigate whether different construals actually correspond to different conceptualizations of the situation.

We investigate whether linguistic encoding affects the way in which events are perceived, and thus potentially conceived, by speakers. We focused on three different linguistic contrasts, ranging from quite obvious to very subtle: preposition (dominant – subordinate), voice (active – passive), and the dative case (noun phrase – prepositional phrase). Sixty University of Sheffield students and staff participated in an eye-tracking study in exchange for 7 GBP. We used an EyeLink Portable Duo eye tracker (SR Research Ltd.).

In block 1 of the experiment, participants viewed a set of 48 full-coloured photographs depicting naturalistic scenes/events; each image was presented for 3500 ms. Across blocks 2 and 3, participants heard 96 individual sentences describing these 48 different events in different ways (e.g., active vs. passive). Each sentence was immediately followed by an image from block 1, depicting the event described in the sentence. Each image contained 2 or 3 interest areas (empirically determined by combining eye-tracker generated heat maps with relevant events described in the corresponding sentences).

We fitted a Generalized Additive Mixed Effects model to the relationship between eye movements and condition (i.e., free viewing vs. different constructions describing the same event). The dependent variables were: (1) dwell time (i.e., summation of the duration across all fixations) on the current interest area; and (2) first fixation time (i.e., start time of the first fixation to enter the current interest area).

Our results suggest that different construals do not necessarily correspond to different conceptualizations of the situation. Namely, construal did not affect the order in which the elements of the scene (e.g., agent, patient) are accessed. On the other hand, different language constructions modulated both the time at which each element is accessed for the first time and the total time spent viewing each element, suggesting different levels of processing.

**References**

- Langacker, R. (1987). *Foundations of cognitive grammar: Vol. 1.* Stanford, CA: Stanford University Press.

### The effect of hyphenation on reading novel words

Tuomo Häikiö & Tinja Luotojärvi

University of Turku, Finland

In Finland, hyphenation is used in reading instruction so that for beginning readers each word is hyphenated at syllable boundaries (e.g., “en-ter”). Hyphens are then gradually removed so that towards the end of 2nd grade only new and long words are hyphenated. Nevertheless, hyphenation at the syllable level slows down reading already during the 1st grade (Häikiö et al., 2015, 2016). This implies that hyphenation forces readers to process words via smaller units than preferred, relying more on the phonology than orthography. In reading new words, phonology is crucial even for skilled readers (Share, 1995), as they cannot rely on the orthographic representation. Since hyphenation highlights the phonology of the word, it may facilitate reading during the first encounter with the word. However, since the orthographic representation is already quite stable after four exposures (e.g., Nation, Angell, & Castles, 2007), this effect is likely to wear off quickly.

The present study examined two main research questions: (1) Does hyphenation facilitate early readers when they encounter new words for the very first time? (2) Does the effect change its nature as the orthographic representation builds?

To assess these questions, Finnish 1st and 2nd graders read stories about animals (16 in total) while their eye movements were registered. Each story introduced an animal and included four occurrences (exposures) of the target word. To be sure that none of the children had no prior exposure to the target words, novel pseudo-words were used. The main manipulation was syllable boundary cue; for each participant, in half of the stories all target words were hyphenated at the syllable level, while in the other half they were not. The hyphenation was counterbalanced between the participants. The other words were never hyphenated. The eye movement measures on the target words were analysed using multiple regression mixed-effects modelling with stepwise backward elimination procedure.

Surprisingly, syllable boundary cue did not interact with exposure. Instead, hyphenated words elicited longer reading times regardless of exposure and grade. As for the other main effects, target words were read faster with increasing exposure, and 2nd graders read target words faster than 1st graders.

The findings replicate those of Häikiö et al. (2015, 2016), but extend them to the first encounter of the word; hyphenation at the syllable level slows down word reading already during 1st grade regardless of whether the word has been seen before or not. Even though hyphenation is connected to the phonological access of the word, it enforces piecemeal processing which is likely to hinder the processes connected to orthographic mapping.

**References**

- Häikiö, T., Bertram, R., & Hyönä, J. (2016). The hyphen as a syllabification cue in reading bisyllabic and multisyllabic words among Finnish 1st and 2nd graders. *Reading and Writing*, 29, 159-182. doi: 10.1007/s11145-015-9584-x
- Häikiö, T., Hyönä, J., & Bertram, R. (2015). The role of syllables in word recognition among beginning Finnish readers: Evidence from eye movements during reading. *Journal of Cognitive Psychology*, 27, 562-577. doi: 10.1080/20445911.2014.982126
- Nation, K., Angell, P., & Castles, A. (2007). Orthographic learning via self-teaching in children learning to read English: Effects of exposure, durability, and context. *Journal of Experimental Child Psychology*, 96, 71-84. doi: 10.1016/j.jecp.2006.06.004
- Share, D. L. (1995). Phonological recoding and self-teaching: Sine qua non of reading acquisition. *Cognition*, 55, 151-218. doi: 10.1016/0010-0277(94)00645-2

### Processing of inflectional stem changes of Finnish words in native speakers and L2 learners

Rosa Salmela¹, Minna Lehtonen¹, Seppo Vainio², & Raymond Bertram²

¹ Åbo Akademi University, Turku, Finland

² University of Turku, Finland

**Background:**

Finnish is a language of rich morphology. In addition to a large number of affixes, there is an abundance of stem alterations that might obscure recognition of the word-internal structure. These characteristics may challenge L2 learners’ lexical processing. In two experiments, we investigated: (a) to what extent morphological complexity and stem changes affect L2 speakers’ word recognition in Finnish; and (b) to what extent these effects are modulated by sentence context.

**Methods:**

Participants were native speakers of Finnish and low- to intermediate-level L2 learners. The target word set consisted of three conditions: monomorphemic nouns, e.g. *lääkäri* (“a doctor”); inflected nouns, e.g. *aamu*: *aamu+lla* (“in the morning”); and inflected nouns with stem alteration, e.g. *ilta*: *illa+lla* (“in the evening”). Experiment 1 used a visual lexical decision task, in which the target words were presented in isolation. In Experiment 2, the same target words were embedded in matched sentence contexts and eye-movement patterns were recorded during reading.

**Results:**

The lexical decision results showed the standard response time delay of inflectional processing in native speakers, and both groups displayed longer RTs for inflected words with stem changes. However, whereas for L1 speakers the error rates were equally low across conditions (1 to 2%), L2 learners made more mistakes with inflections including stem changes than for monomorphemic nouns or transparent inflections. Preliminary results from Experiment 2 suggest that inflected words with stem changes cause delay also in sentence reading particularly in L2 learners, and this effect may vary depending on inflectional case or word length.

**Conclusion:**

The results underline the notion that idiosyncratic language characteristics of Finnish challenge the L2 learner and that these features require extra attention in educational settings.

### The role of spaces in segmenting Finnish and Chinese text

Raymond Bertram¹, Liyuan He², & Simon Liversedge³

¹ University of Turku, Finland

² Tianjin Normal University, China

³ University of Central Lancashire, UK

In alphabetic languages like English, word boundaries are clearly indicated by interword spaces. It has been shown that presenting text in unspaced format slows down reading in English to a great extent (Rayner, Fischer, & Pollatsek, 1998). The main reason is that an efficient visual segmentation cue like the space needs to be replaced by much more subtle and less-practiced segmentation cues: e.g., transitional probabilities between letters. Excluding spaces may also add confusion to the interpretation of compound words: for instance, is the first constituent of “mountain lion” in “hesawthemountainlionfromadistance”) a singular direct object or indeed the first constituent of a biconstituential compound word? In Chinese, text is presented in unspaced format and one of the main tasks is to decide whether subsequent characters need to be unified to form compound words or not. We hypothesized that here the addition of spaces may be helpful in compound resolution.

In Experiment 1, we investigated reading of Finnish spaced vs. unspaced text and showed that in unspaced text: (a) transitional letter sequence probabilities become very important in word boundary detection; and (b) eye movement behaviour is sensitive to the potential ambiguity of compound words. In Experiment 2, Chinese readers read spaced vs. unspaced text including 3-character clusters (ABC) that could be segmented into an AB+C or A+BC two-word combination, with the preceding context guiding the correct interpretation. We found that spacing facilitates reading of the 3-character-clusters, as long as it is in line with the context interpretation. A peculiar finding was that the text was read faster before the ambiguity when unspaced, but faster in the spaced condition after the local ambiguity. Both experiments show that spacing may be facilitative in case of local ambiguity.

**References**

- Rayner, K., Fischer, M. H., & Pollatsek, A. (1998). Unspaced text interferes with both word identification and eye movement control. *Vision Research*, 38(8), 1129-1144.

## SESSION 3: Special populations

### Using different eye-tracking technologies for recognizing oculomotor problems

Ruben Watanabe, Mads Gjerstad Eide, Ilona Heldal, Carsten Helgesen, Atle Geitung, & Gunvor Birkeland Wilhelmsen

Western Norway University of Applied Sciences

Many people struggle with oculomotor issues. Oculomotor difficulties (i.e., problems moving and keeping both eyes focused on the same place) may make it difficult to read and cause headaches, pain around the eyes, or fatigue. These problems have traditionally been identified by manual vision screening or using expensive eye trackers, mainly for scientific use. To have a more affordable application is important, since there are many people with possible oculomotor problems. These problems are congenital, consequences after accidents, or due to some health-related issues such as stroke or brain injury. Also, there are many potential users who would benefit from being tested thoroughly.

Inexpensive eye trackers are now on the market, making this technology available to a wider range of applications and users. We are investigating how, and to what degree, an inexpensive eye tracker can be used for screening to discover oculomotor problems.

The initial motivation for this research was to enable a more systematic screening of children with an inexpensive technology and affordable application. According to research (Wilhelmsen, 2012, p. 9), up to 25% of children have some kind of oculomotor problems which affects their learning in school. In Norway, children are going through a vision screening before starting school and this test is only focused on their distance visual acuity. No systematic testing of oculomotor capabilities is performed, and consequently, many children may be misdiagnosed as having dyslexia or ADHD.

This research presents a solution for recording and visualizing eye movements. Movement of each eye separately is visualized using time plots divided into the horizontal and vertical components, or as an animation showing how each eye moves in relation to the stimuli. The system focuses on reading as well as following moving objects across the screen to assess smooth pursuit and saccadic movement. It is therefore applicable by children and patients who can read and those who cannot read.

To evaluate the data quality of an inexpensive eye tracker, 39 nine-year-old children were screened using eye trackers from two different price ranges. The screening was used with several tasks including reading text and following moving objects. Two eye trackers, one inexpensive device and a considerably costlier one, were used to collect eye-tracking data as the participants performed the tasks once for each eye tracker. Manual screening using traditional methods was also performed. A comparison between the two eye trackers indicates that the inexpensive tracker provides sufficiently precise data to detect intermittent vertical and horizontal misalignments of the eyes, and therefore is able to pinpoint a potential oculomotor problem which may be further investigated by a vision expert.

**References**

- Wilhelmsen, G. B. (2012). *Barns funksjonelle syn. Gir synsvansker som ikke klassifiseres etter ICD-10, behov for tiltak?* (in Norwegian). Bergen, Norway. Høgskolen i Bergen.

### Eye tracking and serious games used for oculomotor training

Håvard Homme Pettersen, Ilona Heldal, Carsten Helgesen, Atle Geitung, & Gunvor Birkeland Wilhelmsen

Western Norway University of Applied Sciences

Many people struggle with eye motoric issues, both children and people with brain damage from disease or accidents. It is possible to improve their vision by training methods, but there is limited research in this field. Most of the training methods used today are physical – e.g., involving real-life physical items like balls, mazes on paper, picture cards, and board games – and a vision expert always needs to be present and participate in the training.

We are investigating how, and to what degree, a computer application may make the training more available and enable users to train partly by themselves using a computer. We present a solution system based on eye tracking and serious games, and covering six different training methods aimed at strengthening various visual deficiencies.

While it has been shown (e.g., Thiagarajan et al., 2014) that oculomotor abilities can be improved by training, there are only a few computer-based solutions built to assist this, and according to our knowledge today, without using eye-tracking technologies; e.g., commercial digital training solutions such as VisionBuilder^1^ or CogPack^2^. These are based on a set of games requiring the user to look at different parts of the screen depending on the needed focus of attention to solve a task in the game. Our application uses eye tracking both to control the application solely through their eye movements, but also as a crucial element in the training process to steer the tasks in the application. Users can follow a moving object, change directions and speed, or pop balloons with their gaze. This provides the methodological basis since the application can be used by people with paralysis or other movement disabilities (Pettersen, 2018).

Studying other games in general, it has been shown that eye tracking can be motivating and fun for the users since their eye motoric abilities are directly connected to the feedback through the application. This may lead to users being more willing to spend time on training, which could lead to faster rehabilitation.

This application is based on recognizing eye movements via a computer program with eye tracking implemented previously (Watanabe et al., 2018). This involves saccades, smooth pursuit, visual attention, and visual memory. However, more research in this field is required for further developing the games and validating the application and investigating how physical training can be completed by the games; e.g., what can be trained in this way and what cannot. At the present stage, such limitations include training that involves peripheral vision or body movements. The application is a newly developed solution and has so far only been evaluated by a few vision experts. Their overall conclusion is positive, and they find the application useful and complementary to the physical training of today.

**References**

- Pettersen, H. H. (2018). Design and development of serious games for oculomotor training using eye tracking. MSc thesis in Software Engineering, Western Norway University of Applied Sciences.
- Thiagarajan, P., & Ciuffreda, K., J. (2014). Versional eye tracking in mild traumatic brain injury (mTBI): Effects of oculomotor training (OMT). *Brain Injury*, 28(7), 930-943. doi: 10.3109/02699052.2014.888761
- Watanabe., R., Eide, M. G., Heldal, I., Helgesen, C., Geitung, A., & Wilhelmsen, G. B. (2018). Using different eye-tracking technologies for recognizing oculomotor problems. *Scandinavian Workshop on Applied Eye Tracking (SWAET 2018)*.

### Concrete vs. abstract processing in Repetitive Negative Thinking: The impact on affect and attentional processes – evidence from an eye-tracking study

Monika Kornacka¹, Céline Douilliez², & Izabela Krejtz¹

¹ SWPS University of Social Sciences and Humanities, Poland

² University of Lille, France

Repetitive Negative Thinking (RNT) is a cognitive process defined as dwelling repetitively on one or more negative concerns and perceived as difficult to control (Ehring & Watkins, 2008). RNT is involved in several psychological disorders such as depression, anxiety, eating disorders, and addictions (Watkins, 2008). However, recent research suggests that RNT is not maladaptive per se, but its adaptive feature depends on processing mode. There are two processing modes in RNT: abstract analytic leading to impaired emotional regulation, and concrete experiential enhancing adaptive emotional regulation (Watkins, 2008).

The repetitive and uncontrollable character of RNT might be imputed to attentional disengagement impairment (Whitmer & Gotlib, 2013). However, there are only few experimental studies exploring this link and none of them distinguishes between adaptive (concrete) and maladaptive (abstract) RNT processing mode. The aim of the current study is to investigate how different RNT processing modes affect attentional disengagement from negative stimuli and focal vs. ambient information processing.

Depressive participants and healthy controls were randomly allocated to one of three processing induction groups (abstract vs. concrete processing vs. distraction condition). First, they underwent RNT activation (Goal Cueing Task; Roberts, Watkins, & Wills, 2013), followed by processing induction. Next, participants were asked to complete six positive and six negative sentences starting with “I am” with a word chosen from a circle composed of words. Each circle contained 15 words (five negative, five positive, and five neutral words, randomly located) and was displayed for 30 seconds. Participants’ eye movements were recorded with an SMI eye tracker (120 Hz). Participants’ affect and state rumination were controlled in between each stage of the experiment (i.e., RNT activation, processing mode induction, and attentional processes measures).

The study is in the stage of data gathering. The analyses will include between-group comparisons of attentional disengagement from negative, positive, and neutral words and changes in ambient-focal attention (Krejtz, et al., 2016). The results will be discussed from the perspective of the hypothesis of attentional disengagement impairment in RNT, but also from a clinical perspective of potential efficiency of attentional trainings in treatments addressing maladaptive RNT.

This study is supported by a POLONEZ 2 (2016/21/P/HS6/04009) grant from the Polish National Research Centre with the founding from the European Union’s Horizon 2020 research and innovation program under the Marie Skłodowska-Curie grant agreement No. 665778 awarded to the first author.

**References**

- Ehring, T., & Watkins, E. R. (2008). Repetitive Negative Thinking as a transdiagnostic process. *International Journal of Cognitive Psychotherapy*, 1(3), 192-205. doi: 10.1177/1073191117693923
- Krejtz K., Duchowski A., Krejtz I., Szarkowska A., & Kopacz A. (2016). Discerning ambient/focal attention with coefficient K. *Transactions on Applied Perception*, 13(3), 1-20.
- Roberts, H., Watkins, E. R., & Wills, A. J. (2013). Cueing an unresolved personal goal causes persistent ruminative self-focus: An experimental evaluation of control theories of rumination. *Journal of Behavior Therapy and Experimental Psychiatry*, 44(4), 449-455. doi: 10.1016/j.jbtep.2013.05.004
- Watkins, E. R. (2008). Constructive and unconstructive repetitive thought. *Psychological Bulletin*, 134(2), 163-206. doi: 10.1037/0033-2909.134.2.163
- Whitmer, A. J., & Gotlib, I. H. (2013). An attentional scope model of rumination. *Psychological Bulletin*, 139(5), 1036-1061. doi: 10.1037/a0030923****

### Eye-tracking control in visual prostheses improves pointing precision

Avi Caspi

Jerusalem College of Technology, Israel

Visual scanning by sighted individuals is achieved using eye and head movements. Conversely, scanning the line-of-sight of the prosthesis is achieved by head movements alone, since eye movements can introduce localization errors. This study demonstrates that a scanning mode utilizing eye movements enhances the performance of visual prosthesis. In this paper, we will present and discuss the technical challenges, and specifically, how to calibrate an eye tracker for blind users.

The integration of an eye tracker in the visual prosthesis allows the measurement of gaze position in real time to adjust the region of interest (ROI) that is sent to the implant within the wide field of view (FOV) of the scene camera. The user will be able to use combined eye-head scanning: shifting the camera by moving their head and shifting the ROI within the FOV by eye movement. Because traditional eye-tracker calibration methods require direct fixation at points in space, this method cannot be used in the blind. We demonstrated that correlating the pupil location at the onset of the stimulation with the head-centred percept location can calibrate and align the eye tracker on Argus II users. Our experimental results with 10 patients show that integrating a calibrated eye tracker reduces the amount of head motion and improves visual stability in Argus II users.

Stimulating the same position in retinotopic coordinates can create percepts at different world-based coordinates, depending on the patient’s gaze position (Sabbah et al., 2014). The perceived location in the world is a function of the location of the electrical stimulation on the retina and the instantaneous position of the eye (Caspi, Roy, Dorn, & Greenberg, 2017). Patients naturally integrate the eye’s position with the retinotopic percept location to localize objects in world coordinates. As a result, integrating an eye tracker into the Argus II to shift the ROI based on eye position for eye-head scanning is feasible, improves pointing precision, and reduces head movements in a localization task.

**Figure 11:**
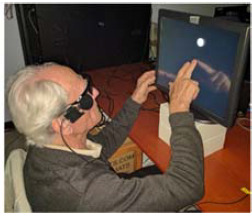
A white target appeared on a touch-screen monitor and the patients were instructed to report the location of the target by touching the monitor. The spread of the responses using combined eye-head vs. head-only scanning were compared.

**Figure 2.**
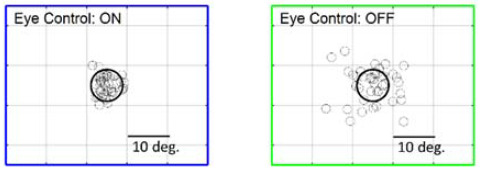
Pointing location in each trial relative to the mean pointing location in one patient. Left panel: Combined eye-head scanning, eye control enabled. Right panel: Head-only, eye control disabled. Six out of eight patients showed a significantly narrower spread.

**References**

- Caspi, A., Roy, A., Dorn, J. D., & Greenberg, R. J. (2017). Retinotopic to spatiotopic mapping in blind patients implanted with the Argus II Retinal Prosthesis. *Invest Ophthalmol Vis Sci*, 58(1), 119-127. doi: 10.1167/iovs.16-20398
- Sabbah, N., Authie, C. N., Sanda, N., Mohand-Said, S., Sahel, J. A., & Safran, A. B. (2014). Importance of eye position on spatial localization in blind subjects wearing an Argus II retinal prosthesis. *Invest Ophthalmol Vis Sci*, 55(12), 8259-8266. doi: 10.1167/iovs.14-15392

## SESSION 4: Eye-tracking measures: Fixations, saccades, microsaccades, and pupil size

### Is the eye-movement field confused about fixations and saccades? A survey among 124 researchers

Roy S. Hessels^1,2^, Diederick C. Niehorster^3,4^, Marcus Nyström^3^, Richard Andersson^5^, & Ignace T. C. Hooge^1^

^1^ Experimental Psychology, Helmholtz Institute, Utrecht University, Netherlands

^2^ Developmental Psychology, Utrecht University, Netherlands

^3^ Humanities Lab, Lund University, Sweden

^4^ Department of Psychology, Lund University, Sweden

^5^ Tobii AB, Stockholm, Sweden

Eye movements have been extensively studied in a wide range of research fields. While new methods such as mobile eye tracking and eye tracking in virtual or augmented realities are emerging quickly, the eye-movement terminology has been revised only scarcely. Has this caused confusion about at least two of the main concepts: fixations and saccades?

In this study, we assessed the definitions of fixations and saccades held in the eye-movement field, by surveying 124 eye-movement researchers. These eye-movement researchers held a variety of definitions of fixations and saccades, of which the breadth seemed even wider than what is reported in the literature. Moreover, these definitions did not seem to be related to researcher background or experience.

We urge researchers to make their definitions more explicit by specifying all the relevant components of the eye movement under investigation: (1) the oculomotor component: e.g. whether the eye moves slow or fast; (2) the functional component: what purposes does the eye movement (or lack thereof) serve; (3) the coordinate system used: relative to what does the eye move; and (4) the computational definition: how is the event represented in the eye-tracker signal. This should enable eye-movement researchers from different fields to have a discussion without misunderstandings.

### Fluctuations in pupil size reflect lack of external attention

Jaana Simola¹, Matias Palva¹, Mahiko Konishi², Satu Palva¹, & Jonathan Smallwood²

¹ Helsinki Institute of Life Science, Neuroscience Center, University of Helsinki, Finland

² Department of Psychology / York Neuroimaging Centre, University of York, UK

Converging evidence indicates that unusually small or large pupil dilations are associated with momentary lapses in attention to the external environment. Large pupil size predicted slow and inaccurate responses in a simple working memory task (Konishi, Brown, Battaglini, & Smallwood, 2017). Moreover, off- compared to on-task states were associated with reductions in the pupil size. Here, we examined whether scale-free fluctuations as evidenced by long range temporal correlations (LRTCs) in pupil size fluctuations are affected by manipulations of the external task focus.

Participants saw sequences of pairs of shapes (i.e., the Non-Targets, NTs) followed by a target stimulus. In the 0-back condition, participants responded which shape matched the presently perceived target shape. The NTs were thus irrelevant to the task allowing for periods when attention was not constrained by the task. In the 1-back condition, participants had to respond depending on which side the target was on the previous trial, and they had to maintain external attention on the NTs. Participants performed two sessions consisting of alternating blocks (between 40 and 120 s) of the 0-back and 1-back conditions. Focus of attention was measured by the task performance and by self-reports collected at the end of the sessions. The same paradigm has been used before and the results of pupil size dynamics preceding the targets are reported in Konishi et al. (2017).

Data from 24 participants (18–22 years, mean age 19.0; 4 males) from the study by Konishi et al. (2017) were re-analysed here. Pupil size data were recorded using an EyeLink 1000 Desktop Mount (SR Research Ltd., Mississauga, ON, Canada) from the participants’ right eye at 250 Hz. We used detrended fluctuation analysis (DFA; see Hardstone et al., 2012) to quantify the LRTCs of pupil size fluctuations (in a 4–40s time window). The LRTC scaling exponents were quantified separately for each block and the exponents for 0-back and 1-back conditions were obtained by averaging the exponents for the corresponding task blocks.

The pupil size fluctuations were stronger in the 0-back relative to the 1-back task as indicated by greater LRTC scaling exponents. Further, the LRTC scaling exponents correlated negatively with the reported levels of detail in thought in the 0-back but not in the 1-back condition. In both tasks, slower response times were associated with increased levels of self-referential thinking. Our data indicated stronger pupil size fluctuations as well as an association between pupil size fluctuations and the form of self-generated thought when attention was less constrained by the ongoing task, suggesting that pupil size fluctuations could be used as an objective marker of the degree of task focus.

**References**

- Hardstone, R., Poil, S.-S., Schiavone, G., Jansen, R., Nikulin, V., Mansvelder, H. D., & Linkenkaer-Hansen, K. (2012). Detrended fluctuation analysis: A scale-free view on neuronal oscillations. *Frontiers in Physiology*, 3, article 450. doi: 10.3389/fphys.2012.00450
- Konishi, M., Brown, K., Battaglini, L., & Smallwood, J. (2017). When attention wanders: Pupillometric signatures of fluctuations in external attention. *Cognition*, 168, 16-26. doi: 10.1016/j.cognition.2017.06.006

### Implicit sequence learning: Reaction time and pupil response as indicators of motor and perceptual learning

Srdan Medimorec, Petar Milin, Adnane Ez-zizi, & Dagmar Divjak

University of Sheffield, UK

Sequence learning represents a fundamental skill that enables individuals to acquire representations of their environment. Previous research has reported evidence of such distributional (or statistical) learning across different domains. Critically, statistical learning involves implicit learning of temporarily ordered patterns, and such procedural learning of regularities also underlies language acquisition. Implicit learning occurs without awareness, without explicit instruction, and with the knowledge being difficult to verbalize (e.g., Turk-Browne, Scholl, Chun, & Johnson, 2009). A commonly used method to study implicit learning is the serial reaction task (SRT; Nissen & Bullemer, 1987). In this task, individuals are presented with a rapid sequence of elements, while learning is measured by reduced response time (RT) across the presentation. In the current study, we set out to elucidate the mechanisms underlying sequence learning using different novel ocular versions of the SRT.

We investigated whether implicit sequence learning necessarily relies on overt eye movements by contrasting conditions where participants either moved their eyes or fixated on a marker during the task (i.e., motor response learning vs. perceptual learning). We used both RT and pupil response indicators to measure learning. Sixty-eight university students and staff participated in an eye-tracking study.

In Experiment 1a, we introduced a rapid version of an oculomotor SRT. Participants (*N* = 35) were instructed to follow a black dot (the target) appearing in one of the four white squares arranged on the screen. Across the four learning blocks (Blocks 1-4), a 12-element sequence (i.e., the target in one of four squares) was presented 20 times (five sequences per block). In Block 5, an interfering sequence was introduced, followed by an additional block with the original sequence. Each target position was preceded by a 500 ms presentation of white squares; the target was presented for 1100 ms. In Experiment 1b, we investigated whether eye movements were necessary for sequence learning. Participants (*N* = 33) were instructed to keep their eyes on the fixation cross in the learning phase (Blocks 1-4), thus effectively suppressing their eye movements. Importantly, target movements were parafoveally visible (the number of repetitions and timing was the same as in Experiment 1a). In Blocks 5 and 6, participants were asked to follow the target. One of these blocks contained the original sequence, while the other block contained an interfering sequence (the order of sequences was counterbalanced across participants).

Our results indicate that implicit knowledge influences performance and prediction generation in both the move and no-move conditions. While implicit learning was detected using both RT and pupil response in the move condition, in the no-move condition only pupil response was sensitive enough to indicate implicit perceptual learning. Finally, we will discuss the implications of our findings for computational models of learning and prediction.

**References**

- Nissen, M. J., & Bullemer, P. (1987). Attentional requirements of learning: Evidence from performance measures. *Cognitive Psychology*, 19(1), 1-32. doi: 10.1016/0010-0285(87)90002-8
- Turk-Browne, N. B., Scholl, B. J., Chun, M. M., & Johnson, M. K. (2009). Neural evidence of statistical learning: Efficient detection of visual regularities without awareness. *Journal of Cognitive Neuroscience*, 21(10), 1934-1945. doi: 10.1162/jocn.2009.21131

## SESSION 5: Reading comprehension in children and adults

### Reading assessment and eye movements during reading in Swedish children

Andrea Strandberg¹, Mattias Nilsson¹, Gustaf Öqvist Seimyr¹, & Per Östberg²

¹ Department of Clinical Neuroscience, Division of Eye and Vision, Karolinska Institute, Stockholm, Sweden

² Department of Clinical Science, Intervention and Technology, Division of Speech Language Pathology, Karolinska Institute

Research during the last decades has demonstrated that eye-tracking methodology is an advantageous tool to study reading, as it offers an online measure of cognitive processing (Rayner, 1998; Blythe, 2014). A substantial amount of eye movement research has resulted in improved understanding of the reading process in skilled adult readers. Considerably fewer studies have examined reading and its development in children (Blythe & Joseph, 2011).

The current study aims to provide descriptive eye movement data from a population-based sample of circa 3,000 Swedish children. Further, test results on letter-RAN, reading speed, and decoding of words/pseudo-words are presented. Finally, the relationship between eye movement parameters and test scores is examined.

Results show that mean fixation duration decrease with increasing age. Saccades remain somewhat stable across the age groups, while regression probability decreases between school years 1 and 2 to even out between school years 2 and 3. Results on letter-RAN, text reading, and decoding of words and pseudo-words demonstrate improved processing speed, reading speed, and decoding with increasing age. The strongest correlation is between average fixation time and reading speed (words per minute) among children in school year 3 (*r* = 0.6).

Results are largely in line with previous findings (Blythe, 2014), with the exception of the stability in saccade amplitude across school year in the present study. Several previous studies have reported a positive correlation between saccade amplitude and age (Rayner, 1998; Blythe & Joseph, 2011). This discrepancy may be a consequence of the selection of properties used to define saccades or an effect of stimuli design. Further analysis may shed light on this matter. To our knowledge, this is the largest study, with regards to number of participants, on children’s eye movements during reading. In fact, it is probably the largest eye movement study ever performed. It also represents a trove of information on early literacy, eye movements, and reading.

**References**

- Blythe, H. I. (2014). Developmental changes in eye movements and visual information encoding associated with learning to read. *Current Directions in Psychological Science*, 23, 201-207. doi: 10.1177/0963721414530145
- Blythe, H. I., & Joseph, H. S. S. L. (2011). Children’s eye movements during reading. In S. P. Liversedge, I. D. Gilchrist, & S. Everling (Eds.), *The Oxford handbook of eye movements*. Oxford, UK: Oxford University Press.
- Rayner, K. (1998). Eye movements in reading and information processing: 20 years of research. *Psychological Bulletin*, 124(3), 372-422.

### Investigating how teachers interact with a visual model of reading development: An eye-tracking study

Pamela Beach, Pamela McDonald, John Kirby, & Jen McConnel

Queen’s University, Ontario, Canada

Teachers continuously seek out learning opportunities to augment their current curriculum content and pedagogical knowledge (Darling-Hammond & Richardson, 2009). While methods such as surveys and interviews offer information about teachers’ attitudes towards learning, they are limited to participants’ recollection of past events. Using eye tracking to document moment-to-moment processes that occur during learning can generate comprehensive data about teachers’ behavioural learning patterns which, in turn, can contribute to more effective instructional approaches and learning support tools.

This exploratory study used eye-tracking technology to investigate the patterns of visual behaviour of experienced elementary teachers and students in a concurrent teacher education programme, while they interacted with a visual model showing key concepts in reading development and instruction called *The Reading Pyramid* (OISE, 2012). *The Reading Pyramid* is a multimedia learning tool that is divided into two main groups: print-related skills (those that promote the ability to recognize words); and language-related skills (those that support the ability to make meaning of text).

Seven experienced teachers and 11 education students from Ontario, Canada, participated in this exploratory study (*N* = 18). Participants were asked to think about how the different literacy components work together to support children’s reading development while they studied *The Reading Pyramid* and corresponding text. Areas of interest (AOIs) on the pyramid and keywords in the text were predetermined. A Tobii Pro X3-120 eye tracker was used to record participants’ eye- movement patterns. The time participants could study the pyramid was open-ended. Prior to studying the pyramid, participants completed a test of prior knowledge about reading development and instruction.

Results show that, on average, experienced teachers spent more time (in seconds) studying the pyramid and corresponding text (*M* = 213.47, *SD* = 62.56) than students (*M* = 162.50, *SD* = 37.22). The experienced teachers also fixated longer on foundational areas of the pyramid devoted to vocabulary *t*(16) = 2.39, *p* = 0.030, text structures *t*(16) = 3.24, *p* = 0.005, and phonics *t*(16) = 3.11, *p* = 0.007. Fixation counts were also significantly different between the two groups. Scan path analysis revealed a direct correspondence between AOIs on the pyramid and keywords in the text when experienced teachers transitioned between the two presentation modes. This is in contrast with the students who were more likely to show an inconsistent skimming pattern.

Correlations between participants’ pretest knowledge and fixation counts and duration suggest that prior knowledge and experience influenced participants’ interaction with the multimedia. For instance, pretest knowledge of phonemic awareness instruction was correlated with participants’ fixation duration on AOIs of the reading pyramid, including phonics (*r* = 0.509, *p* = 0.031) and concepts of print (*r* = 0.560, *p* = 0.016). Overall, findings emphasize the role that prior knowledge and experience (or lack thereof) have on multimedia learning in the context of teacher education.

**References**

- Darling-Hammond, L., & Richardson, N. (2009). Research review/teacher learning: What matters. *Educational Leadership*, 66(5), 46-53.
- Duncan-Howell, J. (2010). Teachers making connections: Online communities as a source of professional learning. *British Journal of Educational Technology*, 41(2), 324-340. doi: 10.1111/j.1467-8535.2009.00953.x
- OISE: Ontario Institute for Studies in Education (2012). The balanced literacy diet: Putting research into practice in the classroom. www.LitDiet.org

### Effects of task instructions and topic signalling on text processing among adult readers using different reading strategies: An eye-tracking study

Dexiang Zhang¹, Jukka Hyönä², Lei Cui¹, Zhaoxia Zhu^1^, Shouxin Li¹

¹ Department of Psychology, Shandong Normal University, Jinan, China

² Department of Psychology, University of Turku, Finland

The purpose of the present study was to examine the effects of task instructions and signalling devices on text processing among adult readers using different reading strategies. In Experiment 1, readers read two multiple-topic expository texts, guided either by a summary task or a sentence verification task. In Experiment 2, readers read a multiple-topic expository text with or without signalling the topic sentences by underlining. Eye-tracking methodology was employed to study individual reading strategies in online text comprehension. First-pass and second-pass reading times were recorded for topic, paragraph-medial, and paragraph-final sentences.

A cluster analysis was performed on the eye movement data to distinguish individual reading strategies (Hyönä, Lorch, & Kaakinen, 2002). The analysis revealed four types of readers in the summary task (Experiment 1): topic structure processors (TSPs), slow linear readers (SLRs), nonselective reviewers (NSRs), and fast linear readers (FLRs). However, only three types of readers emerged in the sentence verification task: SLRs, NSRs, and FLRs. SLRs and NSRs paid extra attention to topic sentences expressing the text’s key contents in the summary task but not in the verification task, while FLRs’ reading was guided by the text’s topic structure in both tasks.

Signalling topic sentences with underlining (Experiment 2) helped linear readers to adopt a structure strategy, whereas TSPs used a structure strategy regardless of signalling devices (see Figure 1). These findings demonstrate that the effects of task instructions and signalling devices are different among readers using distinct strategies.

**Figure. 1:**
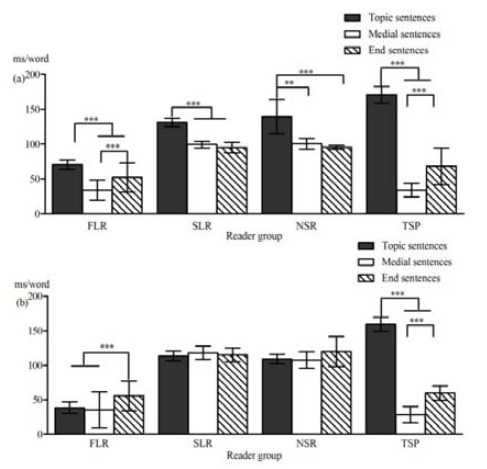
Mean first-pass reading times (mw/word) of the different reader groups as a function of sentence type under the (a) signalling and (b) no signalling conditions in Experiment 2. ** *p* < 0.01, *** *p* < 0.001. FLR = fast linear readers; SLR = slow linear readers; NSR = non-selective reviewers; TSP = topic structure processors.

**References**

- Hyönä, J., Lorch, R. F., & Kaakinen, J. K. (2002). Individual differences in reading to summarize expository text: Evidence from eye fixation patterns. *Journal of Educational Psychology*, 94(1), 44-55.

## SESSION 6: Consumer and gaming behaviour

### Combining screen recordings and eye movement data to analyse consumers’ purchase decisions on dynamic supermarket websites

Kerstin Gidlöf^1^, Nils Holmberg^2,3^, & Annika Wallin^1^

^1^ Cognitive Science, Lund University, Sweden

^2^ Strategic Communication, Lund University, Sweden

^3^ Humanities Lab, Lund University, Sweden

Head-mounted eye-tracking systems such as glasses provide a valuable tool for tracing people’s cognitive processes while performing shopping decisions in physical supermarkets. Since online grocery shopping is steadily increasing in Scandinavian countries, including Sweden, remote eye-tracking equipment could potentially provide a measurement technique with a high level of ecological validity for such screen-based environments. However, due to the dynamic nature of modern websites, where more content is continually loaded into the web page as the user scrolls further down (cf. “infinite scroll”), calculating which web page objects coincide with the user’s visual point of regard has become increasingly difficult. Standard stimulus presentation programs such as SMI Experiment Center are limited to web pages with a static layout. In order to get a better understanding of people’s shopping decisions in online grocery stores, and to develop methods allowing us to compare shopping behaviour across physical and online shopping environments, the current study recorded eye movements and screen activity from 60 participants while they completed three food purchasing tasks on a dynamic supermarket website (www.ica.se).

When entering into the study, each participant received a voucher for 100 SEK to cover the expenses for the food items that they purchased. Participants were seated comfortably in front of a computer monitor with a resolution of 1680 x 1050 pixels, with an SMI RED-m eye tracker mounted at the bottom. A five-point calibration including validation was then performed, and the average measured accuracy during calibration was below 0.5 degrees error both vertically and horizontally (calibration points with an error over 1 degree were never accepted but prompted recalibration). The participants were instructed to buy one food item from three product categories: pasta, cereal, and yogurt. Data collection was handled using the website stimuli presentation available in SMI Experiment Center, which allowed for 10 Hz website screen recording, and eye movements were recorded binocularly at 120 Hz using the SMI iView X software. Consumers also filled out a questionnaire regarding their food preferences.

Screen recordings were first analysed through manual encoding of purchase intervals, which started when participants entered web pages containing any of the product categories investigated (pasta, cereal, and yoghurt). By splitting these video intervals into frames, a complete web page spanning the entire height of all products listed was constructed for each category. These complete web pages were used to calculate the relative visual saliency of all products listed within each category, as well as the vertical position of each product. The onset time of the purchase intervals were used to locate the corresponding eye movement data, and scroll trigger messages in the data were used to perform scroll compensation on the recorded point of regard. Having a complete web page for each product category and scroll compensated eye movement data, an area of interest (AOI) analysis was performed in order to calculate sample count, number of revisits, and whether a product AOI had been visible on screen. In accordance with our expectations, vertical position on the web page had a significantly negative effect on dwell time. Based on previous research on visual attention in physical supermarkets, we expected that visual saliency of online products would have a positive effect on the amount of visual attention.

### Playing profiles in a mathematics game based on eye movement and game log data

Diana-Elena Gratie, Marjaana Puurtinen, & Erno Lehtinen

University of Turku, Finland

Educational games are employed as pedagogical tools to improve students’ engagement and outcomes with mathematics, but so far there is little research to support their most productive use. Understanding the intuitive strategies of players is important for enhancing students’ adaptive strategies during game play. The current study identifies profiles of students freely engaging with a mathematics game (the Number Navigation Game, NNG; see Lehtinen et al., 2015) based on game log and eye-gaze data. The NNG was developed as an environment where students can develop mathematical skills by exploring and practising more advanced arithmetic strategies. The game environment is a map of land and sea with numbers 1-100 superimposed on the sea parts. The player collects resources on the map by performing arithmetic operations that move a boat from its current number location to the number that is the result of the performed arithmetic operation (unless that direct route is blocked by an island). The log file contains all calculations performed. We hypothesized that the list of highly fixated numbers contains the numbers actually used in calculations and thus found in the log files, but that they may also contain numbers that reflect the alternative routes the players considered. The similarities or dissimilarities of the two data sets might reflect different player profiles.

A total of 23 students (with either normal or corrected-to-normal vision) from two universities in Finland took part in the study. They played the NNG with no time constraints while being tracked with a Tobii T60XL eye tracker. An area of interest (AOI) grid was overlaid on the map, with each AOI identifying one number. Based on the total dwell time on each AOI, we computed the percentage of time spent on each visited number with respect to the total time spent on fixating the map and obtained the list of highly visited numbers (above average).

Preliminary findings indicate that some participants indeed used a more directed strategy for searching after a route (profile 1). For these participants, the numbers that they fixate most are precisely the ones they use. Other participants had a considerably larger pool of fixated numbers, including ones that were not used in the calculations (profile 2). For another group of participants, the list of highly visited numbers did not include all numbers in the log file (profile 3). In connection, incidental “wrong” moves (either assuming a wrong outcome or selecting a wrong operation by accident) were determined by identifying numbers that appear in the log file but whose corresponding AOIs were not fixated at all or were fixated extremely briefly. Overall, the eye-movement data brought forth the large differences among all participants: between the participant that visited the fewest numbers and the one that visited the most, there was a difference of 41-46 visited numbers. These profiles will be used for predicting solving strategies in the game. Only 36% of the directed strategy routes were optimal, and of the identified optimal routes only 20% resulted from directed strategies, suggesting that directed search should be combined with strategic identification of alternative routes.

**References**

- Lehtinen, E., Brezovszky, B., Rodríguez-Aflecht, G., Lehtinen, H., Hannula-Sormunen, M. M., McMullen, J., … Jaakkola, T. (2015). Number Navigation Game (NNG): Design principles and game description. In J. Torbeyns, E. Lehtinen, & J. Elen (Eds.), *Describing and studying domain-specific serious games advances in game-based learning*. (pp. 45-61). New York, NY: Springer.

## SESSION 7: Social and temporal illusions

### Seeing is believing? The implied social presence experiment

Gijs A. Holleman^1,2^, Roy S. Hessels^1,2^, Chantal Kemner^1,2,3^, & Ignace T. C. Hooge^1^

^1^ Experimental psychology, Helmholtz Institute, Utrecht University, Netherlands ^2^ Developmental psychology, Utrecht University, Netherlands ^3^ Brain Center Rudolf Magnus, University Medical Center Utrecht, Netherlands

**Background:**

In this study, we investigated how “implied social presence” – the belief that one is being watched by another person ­– influences social perception. Specifically, we were interested in how social presence affects gaze behaviour to the eyes.

**Method:**

A total of 82 participants received one of two instructions, either that they would see a person via a live video connection (“live instruction”), or that they would see a pre-recorded clip (“pre-recorded instruction”). Prior to the experiment, a confederate walked into a separate room to suggest that (s)he was positioned behind a webcam. In fact, regardless of the instructions, all participants were presented with a pre-recorded clip of a person (one of four confederates present at the scene). Participants’ eye movements were recorded using a high-end eye tracker (Tobii TX-300). Afterwards, participants were asked to ignore the instruction and respond whether they thought that the presentation was live or not, and why.

**Results:**

46.3% of the participants responded “live presentation”. Also, the subjective responses suggest that participants used different cues to judge whether the person on the screen was live or not. On the entire dataset (*N* = 82), analyses of eye movements revealed that participants who received the “live instruction” gazed significantly less at the eyes compared to participants who received the “pre-recorded instruction”, indicating an effect of social presence. However, after removing participants (*N* = 14) based on data quality measures (e.g., % data loss, sample-to-sample RMS-deviation), there was no significant difference between groups (*p* = 0.065).

**Conclusion:**

Our study shows that participants can be successfully led to believe that one is engaged with a “live person”. This is highly relevant to researchers interested in the influence of (implied) social presence on, for example, social perception and social gaze. Importantly, when looking at the eye-tracking data (e.g., % total dwell time at the eyes), we found that statistically significant differences between groups were dependent on several data quality criteria. This highlights the crucial importance of data quality for the interpretation of results in eye-tracking research.

### Saccadic chronostasis during sequential eye movements

Supriya Ray, Ankit Mourya, & Ravindra Sahu

Centre of Behavioural and Cognitive Sciences, University of Allahabad, India

Most of our routine activities are visually guided: for example, limb movement follows rapid saccadic eye movements in the direction of an object of interest. To accomplish an action, often such movements are serially ordered in a time-critical fashion. “Saccadic chronostasis” refers to the subjective perception of temporal dilation of the duration between visual events following a saccade (Yarrow, 2010). Although saccadic chronostasis is a robust phenomenon in a single-saccade condition, it remains unclear whether the chronostasis occurs during consecutive saccades as well. We recorded the eye movements of young healthy humans in a pair of modified double-step tasks (Ray et al., 2004). In the “follow task”, subjects directed their gaze to a timer clock either directly (no-step trial) or after a saccade to an intermediate identical object (step trial). In the “redirect task”, subjects inhibited the saccade to the initial target, and directed gaze to the final one. In both tasks, step and no-step trials were randomly interleaved. The clock at the final target location always ticked immediately after the first saccade onset. At the end of each trial, subjects reported the duration of the tick-delay in reference to the offset-delay of flickers presented at the fixation location prior to saccade(s). We found that saccadic chronostasis occurred even in the sequential-saccade condition, although with reduced magnitude, which may be accounted for by predictive covert shift of attention to the final saccade-target. We also observed modulation in the perceived temporal dilation during error correction. Taken together, our data suggest that the saccadic chronostasis is not limited to an isolated eye movement and is potentially influenced by the cognitive context.

**Figure. 1:**
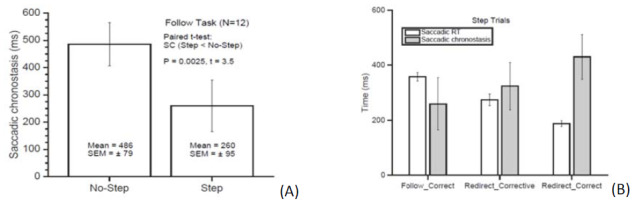
(A) Magnitude of saccadic chronostasis in the follow task during one saccade (no-step) and two saccades (step). (B) Saccadic response time and chronostasis magnitude during consecutive saccades are compared in different cognitive contexts.

**References**

- Ray, S., Schall, J. D., & Murthy, A. (2004). Programming of double-step saccade sequences: Modulation by cognitive control. *Vision Research*, 44(23), 2707-2718. doi: 10.1016/j.visres.2004.05.029
- Yarrow, K. (2010). Temporal dilation: The chronostasis illusion and spatial attention. In A. C. Nobre, & J. T. Coull (Eds.), *Attention and Time* (pp. 137-150). Oxford, UK: Oxford University Press.

## SESSION 8: Film subtitles and musical notation

### Where do subtitlers look? Split attention in the intralingual subtitling process

Anke Tardel¹, Silke Gutermuth¹, Moritz Schaeffer¹, Silvia Hansen-Schirra¹,

Volker Denkel², & Miriam Hagmann-Schlatterbeck²

¹ Johannes Gutenberg University Mainz, Germany

² ZDF Digital Medienproduktion GmbH

While the reception of subtitles has been the subject of various eye-tracking studies (e.g. Bisson et al., 2014; Kruger et al., 2014; Fox, 2018), the process of subtitling has yet to be investigated with established methods from translation process research such as eye tracking and keylogging. Künzli (2017) did an extensive survey among subtitlers on subtitle production, but the process itself was not analysed empirically.

Within Compass^3^, a project that aims at developing an innovative platform for multilingual subtitling, we present results from a usability study of intralingual subtitling. Using a mixed methods approach with eye tracking and keylogging, this study investigates the cognitive load and split attention of subtitlers using FAB Subtitler, a market leader in commercial subtitling tools. The tool includes common subtitle features such as spotting editor, video player with subtitle overlay, a subtitle length and reading speed monitor, as well as an audio track. Four experienced subtitlers from ZDF Digital and four student subtitlers create intralingual subtitles of three 5-minute snippets from the German ZDF documentary series *Terra X* following the Timed Text Style Guide by one of the leading video-on-demand platforms (Netflix). Recording sessions take place in the participants’ usual work setup, last about 1 hour, including recalibrations to ensure data quality, and participants complete a questionnaire regarding their use of the subtitling tool. For quality annotation, all final subtitle files go through quality assurance.

The aim of this study is to find bottlenecks in the subtitling process and to analyse where participants work efficiently (e.g., using shortcuts), or where they lose time and make errors due to split attention on too many tasks simultaneously (e.g., listening to audio, typing, adapting and spotting subtitles). Cognitive load and efficiency in the process is estimated with established measures: i.e., fixation durations and count, regression paths and revisions, pauses and duration, and questionnaire data. We test hypotheses regarding split attention similar to that of online revision compared to final revision during the translation process (cf. Hansen-Schirra et al., in press). We expect long fixations on the bar indicating optimal subtitle duration, and long pauses when subtitlers struggle with adjusting subtitles to shot changes or fast-paced audio; i.e., no one-to-one subtitle possible. This study could reveal subtitling such as first typing and spotting draft subtitles to later fine-tune them in contrast to doing everything in one step. Data collection will be finished in June which gives plenty of time for analysis to present the results in August. Based on the results a new tool will be developed and tested to compare cognitive load and efficiency of both tools.

**References**

- Bisson, M.-J., Van Heuven, W., Conklin, K., & Tunney, R. (2014). Processing of native and foreign language subtitles in films: An eye tracking study. *Applied Psycholinguistics*, 35(2), 399-418.
- Fox, W. (2018). Can integrated titles improve the viewing experience? *Language Science Press*. doi: 10.5281/zenodo.1180721
- Kruger, J.-L., & Steyn F. (2014). Subtitles and eye tracking: Reading and performance. *Reading Research Quarterly*, 49(1), 105-120.
- Künzli, A. (2017). Die Untertitelung – von der Produktion zur Rezeption (Vol. 90). Frank & Timme GmbH.
- Hansen-Schirra, S., Hoffmann, S., Schaeffer, M., & Tardel, A. (in press). Cognitive effort and efficiency in translation revision. In E. Huertas Barros, S. Vandepitte, & E. I. Fernández (Eds.), *Quality assurance and assessment practices in translation and interpreting*.

## POSTER SESSION: Eye-tracking methodology

### Microsaccade detection using pupil and corneal reflection signals

Diederick C. Niehorster^1,2^ & Marcus Nyström^1^

^1^ Humanities Lab, Lund University, Sweden

^2^ Department of Psychology, Lund University, Sweden

In contemporary research, microsaccade detection is typically performed using the calibrated gaze-velocity signal acquired from a video-based eye tracker. To generate this signal, the pupil and corneal reflection (CR) signals are subtracted from each other, a calibration mapping is applied, and a differentiation filter is applied, each of which may prevent small microsaccades from being detected due to signal distortion and noise amplification introduced by these processing steps. We propose a new algorithm where microsaccades are detected directly from uncalibrated pupil and CR signals. It is based on detrending the pupil and CR signals, followed by windowed crosscorrelation of these detrended signals. When tested on 1000 Hz binocular data acquired with an EyeLink 1000 Plus, the proposed algorithm outperforms the most commonly used algorithm in the field (Engbert & Kliegl, 2003), in particular for small amplitude microsaccades that are difficult to see in the velocity signal even with the naked eye. We argue that it is advantageous to consider the most basic output of the eye tracker (i.e., pupil and CR signals), and introduce as little processing as possible when detecting small microsaccades.

**References**

- Engbert, R., & Kliegl, R. (2003). Microsaccades uncover the orientation of covert attention. *Vision Research*, 43(9), 1035-1045.

### Hybrid dwell time and dwell free keyboard

Tanya Bafna & John Paulin Hansen

Technical University of Denmark

Text entry with eye-tracking keyboards (i.e., “gaze typing”) can be performed via two techniques: using dwell-time to select each character and dwell-free methods to predict the characters and words. While dwell-time typing allows the user to choose the exact characters to type and avoid the *Midas Touch* effect, the typing speed is limited by the dwell-time (Liu, Lee, & McKeown, 2016). On the other hand, dwell-free methods commonly use language models to predict the word based on gestures (Wobbrock, Sawyer, & Duchowski, 2008) or probabilities (Ward, Blackwell, & MacKay, 2000). Combining both dwell-time and dwell-free methods, so as to complement one another, could greatly increase the typing speed and aid the prediction method.

The project, still in an initial phase, is built on an open-source assistive keyboard – Optikey (https://github.com/OptiKey/OptiKey/wiki). An exceptional feature used in the keyboard is multikey selection. Using this feature, the user fixates on the first letter of the word for the specified dwell-time, gazes through the rest of the letters without spending time on each of them, and then dwells on the last letter for the dwell-time. There is visual as well as auditory feedback on selection of the first and the last letters.

During the experiment, participants type four sentences using either multi-key selection or dwell-time typing. The Tobii 4C system is used for eye tracking in combination with the Optikey keyboard. The dwell-time is initiated to 800 ms but is adjustable by the participants, which is an attempt to remove bias in the performance of the two methods. Results of comparison between multi-key selection and dwell-time typing in terms of ease of use, error rate, and word-per-minute will be presented at the workshop. We will also include gaze data on pupil and gaze behaviour, in particular the number of read text events per character typed as a measure of uncertainty during typing.

**References**

- Liu, Y., Lee, B. S., & McKeown, M. J. (2016). Robust eye-based dwell-free typing. *International Journal of Human-Computer Interaction*, 32(9), 682-694. doi: 10.1080/10447318.2016.1186307
- Ward, D. J., Blackwell, A. F., & MacKay, D. J. C. (2000). Dasher – a data entry interface using continuous gestures and language models. In *Proceedings of the 13th annual ACM symposium on User interface software and technology – UIST ’00* (pp. 129-137). doi: 10.1145/354401.354427
- Wobbrock, J. O., Rubinstein, J., Sawyer, M. W., & Duchowski, A. T. (2008). Longitudinal evaluation of discrete consecutive gaze gestures for text entry. In *Proceedings of the 2008 symposium on Eye tracking research & applications – ETRA’08* (pp. 11-18). doi: 10.1145/1344471.1344475

### Impact of task complexity on driving a gaze-controlled telerobot

Guangtao Zhang, Katsumi Minakata, Alexandre Alapetite, Zhongyu Wang, Martin Thomsen, &

John Paulin Hansen

Technical University of Denmark

Robotic telepresence systems promote social interaction between geographically dispersed people. Gaze interaction is regarded as a common control mode for severely paralyzed people (Minakata et al., 2018). Gaze interaction with telerobots provides a new opportunity for people with limited mobility. The possibility of gaze-controlled, floor-driving robots has been shown in a prior study (Tall et al., 2009). The quality of eye tracking has been shown to be sufficient for gaze interaction in a bed scenario (Hansen et al., 2011). Situation awareness (SA) plays an important part in telepresence and a high level of understanding of the environment the telerobot is navigating through must be provided (Endsley, 2000). SA is also a primary basis for performance (Endsley, 1995). However, for this kind of gaze-controlled telepresence, it is still unclear how task complexity impacts users’ performance and their SA. Thus, the main research question of this study is: what is the impact of task complexity when driving a gaze-controlled telerobot with a virtual reality head-mounted display (VR HMD)?

A total of 10 participants took part in our experiment (five with a low-complexity task vs. five with a high-complexity task). The dependent variables of interest were, eye movements, position of telerobot, and correctness of answers about information collected during the test. A subjective measure was also collected on experience of comfort and fun. A VR HMD with gaze tracking was provided for each test person to control a robot that carries a 360-degree video camera. The two groups of participants were asked to drive the gaze-controlled robot along two pre-set paths with different complexities. Following the driving test, each participant was interviewed.

With log data and screen recordings captured during the experiments, our analysis results include users’ eye movement behaviours, telerobots’ deviation from pre-set paths, number of collisions, and accuracy of answers about information collected during the test. We present out findings in terms of differences between the two groups.

**References**

- Endsley, M. R. (1995). Measurement of situation awareness in dynamic systems. *Human Factors*, 37, 65-84.
- Endsley, M. R. (2000). Direct measurement of situation awareness: Validity and use of SAGAT. *Situation Awareness Analysis and Measurement*, 10, 1-21.
- Hansen, J. P., Agustin, J. S., & Skovsgaard, H. (2011). Gaze interaction from bed. In *Proceedings of the 1st Conference on Novel Gaze-Controlled Applications – NGCA’11*.
- Minakata, K., Thomsen, M., & Hansen, J. P. (2018). Bicycles and wheelchairs for locomotion control of a simulated telerobot supported by gaze- and head-interaction. In *Proceedings of the 11th PErvasive Technologies Related to Assistive Environments Conference* (pp. 371-378).
- Tall, M., Alapetite, A., San Agustin, J., Skovsgaard, H. H., Hansen, J. P., Hansen, D. W., & Møllenbach, E. (2009). Gaze-controlled driving. In *Extended Abstracts on Human Factors in Computing Systems – CHI’09* (pp. 4387-4392).

## POSTER SESSION: Language processing and reading comprehension

### Reading assessment of children with reading disabilities using scanpaths

Elena Haffmans & Søren Søbæk Petersen

University of Copenhagen, Denmark

In Denmark, children with reading difficulties are provided special reading training in small groups or individually. A new reading application records the eye movements of the child as they read aloud to assist the teacher in monitoring their reading strategy. Analysing scanpaths of these readings, we try to detect passages of fluent reading. The predictiveness of the scanpath measure is validated against common text difficulty measures. The teachers’ markings of misread words are used as a gold standard in our setup. Automatically discriminating fluent from non-fluent reading can be used as, e.g., automatic reading assistance and evaluation.

The use of scanpaths in readers with reading disabilities is an underexplored area of research. Contemporary eye-tracking research mostly focuses on only different fixation measures. By looking at the whole scanpath, in which the total of tracked x- and y-coordinates are included, we obtain a richer representation of individual readings. Scanpaths have been used in reading research, but before Malsburg, Kliegl, and Vasishth (2015), they have not been employed as a sentence-based measure to distinguish between different readings. The goal of the present work is to check whether we can employ scanpaths as a discriminator to differentiate easy and difficult readings.

Data is gathered in a classroom setting and consists of over 9000 sentences read by 71 Danish children with a reading difficulty. We adopt Malsburg et al.’s scanpath similarity measure to compare similar sentences read by these children. This enables us to look at the variance between different readings of similar sentences, some read correctly and some with words marked as misread by the teachers. We also analyse scanpath regularity through Recurrence Quantification Analysis.

The misread markings are then contrasted to different sentence difficulty measures, including surprisal cost and phonetic ambiguity, by using correlation measures. This comparison enables us to give an indication of the quality of a new reading, controlling for the difficulty of the sentence.

**Figure. 1:**
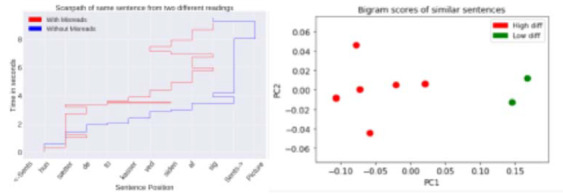
(a) Two scan path plots of the same sentence, one with at least one misread word (red) and without any misreads (blue). “<- Sents” means fixation on a previous sentence on the same page, “Sents ->” refers to looking at a sentence ahead on the same page. “Picture” means the reader looked at the image on the same page. (b) shows the similarity between different readings of similar sentences, meaning they have the same sentence length and similar word lengths. One point is one reading. The clusters are based on the surprisal (bigram) score of each read sentence. PC1 and PC2 are the principal components.

**References**

- Malsburg, T., Kliegl, R., & Vasishth, S. (2015). Determinants of scanpath regularity in reading. *Cognitive Science*, 39(7), 1675-1703. doi: 10.1111/cogs.12208

### Using naturalistic eye-tracking data to understand children’s reading difficulties

Maria Barrett¹, Joachim Bingel¹, Sigrid Klerke², & Laura Winther Balling³

¹ University of Copenhagen, Denmark

² EyeJustRead, Denmark

³ Copenhagen Business School, Denmark

Eye-tracking studies of reading primarily involve skilled adult readers in controlled laboratory settings. Before this research can contribute to practical reading teaching, it is necessary to verify that insights from this domain generalize to the settings and populations where reading teaching happens. Owing to the developments in eye-tracker price and quality, gaze recordings from naturalistic reading teaching can be leveraged for this purpose and for developing models for automated reading performance analysis.

In the present research, we use eye-tracking data collected during real reading training sessions in Danish schools where 95 students, who had been referred to extra reading intervention, read texts of varying difficulty aloud while their eye movements were recorded. The data was analysed in two ways.

The first analysis investigated students’ total gaze time per word in a linear mixed model. The largest effects were for word length, with longer gaze times for longer words, and word frequency, with particularly large facilitatory frequency effects for those words that occurred for the first time in the given text. These effects are similar to what we observed with adult, skilled readers, but with generally larger effect sizes in the present study. In addition, the analysis showed significant effects of the word’s position in the line and text, and the readers’ experience with the system, both within and across sessions.

Secondly, the data was analysed to predict misread words using machine-learning techniques. Automatic identification of misread words may be useful for semi-automating reading assessment and assistance. In addition to 16 basic features, we explored the contribution of three feature groups: 7 linguistic features, 15 word-level gaze features, and 8 context-level gaze features. Combining context- and word-level gaze features gave the best result with 41.19 F1 score.^4^ Using only the basic features gave an F1 score of 18.78.

In sum, these analyses show that eye tracking in a naturalistic reading teaching setup shows a range of gaze behaviours that are also observed in laboratory-based research on eye movements in reading, and that this data may be leveraged for developing automatic analysis of reading. This demonstrates the possibility for eye tracking to contribute directly to practical reading teaching by helping teachers identify and support students’ development of reading strategies. Further avenues of research include closer investigation of students’ individual profiles, the role of word context, and individual automatic reading assessment and feedback.

### Reading, decoding, and eye movements: A neuropsychological assessment of eight students in grade 3

Anna Carin Gran Ekstrand, Mattias Nilsson Benfatto, & Gustaf Öqvist

Marianne Bernadotte Centrum, Karolinska Institute, Stockholm, Sweden

Reading ability is a prerequisite for academic success and a key factor in the development of mental health. Early identification of individuals at risk is therefore essential. The purpose of this study was to examine the relationship between deviant measurements from an eye-movement screening tool in reading and the underlying cognitive functions through neuropsychological assessment.

In total, eight individuals in grade 3 participated. They had been randomly selected from a group defined as “risk for dyslexia” in a larger project at the Marianne Bernadotte Centre, Karolinska Institute. The purpose of this larger project was to develop a screening instrument for early identification of children at risk for dyslexia by combining eye tracking with machine learning.

The results support earlier research findings which suggest that deviant measurements from an eye-movement screening tool in reading is a first effective step in finding the students at risk for reading and decoding difficulties. The results also show a possibility of other cognitive domains being affected such as verbal working memory, verbal executive functions, attention/divided attention, psychomotoric speed, and rapid naming (Figure 1). Due to significant differences both intra- and inter-individually, two conclusions are drawn. The first concerns the importance of completing a more thorough assessment of different reading abilities to reach a deeper understanding of the specific individual. The second concerns the importance of including other cognitive domains in the assessment to reach a more nuanced knowledge and understanding of individual’s cognitive strengths and weaknesses. The cognitive weaknesses may not be related directly to reading and decoding abilities per se, but they still can have a negative impact on reading development, as well as on learning at large, concentration, and attention. A deeper and more nuanced understanding will also be of great importance in the development of individually and successfully designed interventions.

**Figure. 1:**
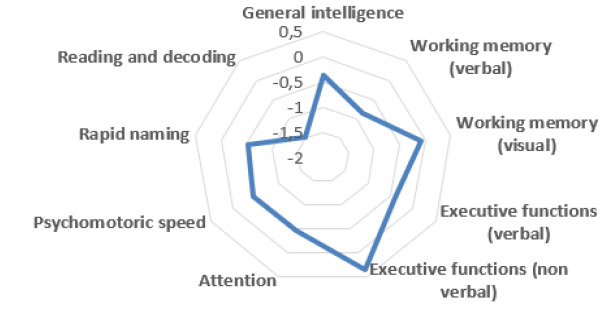
Results on cognitive domains

**References**

- Rello, L., & Ballestreos, M. (2015). Detecting readers with dyslexia using machine learning with eye tracking measures. In *Proceedings of the 12^th^ Web for all Conference* (p. 16).
- Benfatto, M. N., Seimyr, G. Ö., Ygge, J., Pansell, T., Rydberg, A., & Jacobsen, C. (2016). Screening for dyslexia using eye tracking during reading. *PLoS ONE*, 11(12), e0165508. doi: 10.1371/journal.pone.0165508
- Reichle, E. D., Liversedge, S. P., Drieghe, D., Blythe, H. I., Joseph, H. S., White, S. J., & Rayner, K. (2013). *Using EZ Reader to examine the concurrent development of eye-movement control and reading skill*. *Developmental Review*, 33(2), 110-149.
- Rayner, K. (1998). Eye movements in reading and information processing: 20 years of research. *Psychological Bulletin*, 124(3), 372-422.

### Split screen exploration in sign language users: An eye-tracking study

Olga Soler Vilageliu, Marta Bosch Baliarda, & Pilar Orero

Transmedia Catalonia Group, Autonomous University of Barcelona, Spain

In this research, we applied eye-tracking measures to examine how sign language users explore split TV screens. We used a sign-translated documentary where both visual and linguistic information is relevant. Four possible screen combinations (see Figure 1) resulted from combining Position of the SLI sub-screen (Left/Right) with Size (Small = 1/5 of the screen width; Medium = 1/4 of the screen width).

**Figure. 1:**
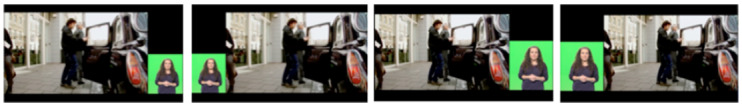
Screen compositions, from left to right: Small/Right; Small/Left; Medium/Right; Medium/Left

Participants were 28 deaf signers from 17 to 74 years old. The documentary *Joining the Dots* (Romero-Fresco, 2012) was translated into Catalan SL and edited into four clips displaying all four combinations. All participants watched all contents in different combinations using a Latin Square design, while eye movements were recorded with a Tobii eye tracker. We defined two areas of interest: SLI sub-screen and documentary sub-screen. After watching each clip, participants filled up two questionnaires to evaluate their recall of linguistic content (SL interpretation) and visual content (Documentary visual information).

We analysed the effects of the factors Size, Position, and Area on the measures Fixation Count, Fixation Duration, and Total Visit Duration using a GLM with repeated measures. Area was the only factor showing significant effects: the SLI sub-screen was visited for a longer time, with longer fixations, and more fixations. Position and Size in this experiment were not relevant for sign language users, whose pattern of exploration consists mainly on focussing in SLI with shorter gazes to the general screen.

We ran paired samples T-tests in order to check if there were differences between linguistic and visual recall for each screen configuration. Linguistic recall was better for the Small/Left configuration. Visual recall did not differ significantly from linguistic recall, even if users tended to make longer visits with longer fixation durations on the SLI sub-screen. Probably deaf sign language users collect visual information parafoveally. This interpretation is based on some perceptual studies that point out that parafoveal vision is enhanced in sign language users (Dye, Seymour, & Hauser, 2016; Siple, 1978).

A tentative conclusion from our results is that sign language users seem to adapt swiftly to different screen configurations. Further studies could test on other screen designs to favour usability and guide directions to content producers. This study was sponsored by the Project grant FFI2015-64038-P, MINECO/FEDER, UE.

**References**

- Dye, M. W., Seymour, J. L., & Hauser, P.C. (2016). Response bias reveals enhanced attention to inferior visual field in signers of American Sign Language. *Experimental Brain Research*, 234, 1067-1076. doi: 10.1007/s00221-015-4530-3
- Romero-Fresco, P. (2012). *Joining the Dots*. https://vimeo.com/51675746
- Siple, P. (1978). Visual constraints for sign language communication. *Sign Language Studies*, 19, 95-110. doi: 10.1353/sls.1978.0010

### Perceptual priming and syntactic choice in the Russian language: A multimodal study

Mikhail Pokhoday¹ & Andriy Myachykov²

¹ Higher School of Economics, Moscow, Russia ²Northumbria University, Newcastle, UK

In a fully developed production system, perception provides an input of information about the event, attention foregrounds relevant/important information for the conceptual analysis, and subsequent language production mechanisms collaborate to generate speech (Levelt, 1989). A part of this complex process is the necessity to select between simultaneously available syntactic alternatives. For example, English language provides several options that can describe the same visual event: e.g., an officer chasing a burglar. These minimally include: (1) “The officer is chasing the burglar”; and (2) “The burglar is (being) chased by the officer”. These active- and passive-voice alternatives differ in assigning object and subject roles to agent (officer) and patient (burglar). Existing evidence suggests that the system responsible for assigning the grammatical roles is sensitive to the distribution of the speaker’s attention within the described scene (see Tomlin & Myachykov, 2015, for a recent review). Specifically, a speaker of English is more likely to choose a passive-voice frame when her attention is directed to the patient of the described event and she is more likely to use an active-voice frame when the agent is in her attentional focus (e.g., Myachykov, et al., 2012). While this and other studies indicate a regular interplay between attention and syntactic choice, they also exclusively used variants of the visual cueing paradigm (Posner, 1980). As a result, the reported link between attention and syntactic choice cannot be generalised beyond the visual modality. A more ecologically valid proposal needs to take into account the multi-modal nature of attention.

Here, we report results of a series of sentence production experiments, in which Russian native speakers described visually presented transitive events – e.g., “kick” (“pinat”), “chase” (“presledovat”/ “ubegat”) – while being recorded with an eye tracker. In these experiments, we have recorded eye-tracking data using an EyeLink 1000+ system. We used eye tracking to control eye position during fixation screen as well as on presentation of the stimuli pictures. To control attention allocation prior to attention manipulation, we implemented a fixation trigger in the experimental protocol. Thus, participants have been presented with a cue only after prolonged fixations in the centre of the screen. Also, we assessed the effectiveness of the marker based on the amount of first fixations on the marked referent.

In half of the trials, the agent appeared on the left and in the other half – on the right. Speakers’ attention to the referents was manipulated by means of lateral cues. In Experiment 1 by visual cue (a red circle); in Experiment 2 – auditory (beep played monaurally); in Experiment 3 – motor (participants were prompted to press a left or a right key depending on the colour of the central fixation cross). Hence, the Cued Referent (Agent/Patient) was crossed with the Cue Type (Visual, Auditory, Motor). The proportion of the sentences where the cued patient referent was put in the sentence before agent was the dependent variable. In Experiment 1, we registered a main effect of visual cue location – patient has been chosen as a starting point in the sentence more often when he had been cued: X2(1) = 4.15, *p* = 0.042. Also, there was a main effect of event orientation – Russian speakers produced more patient-first sentences when the patient was on the left in the picture: X2(1) = 3.91, *p* = 0.048. There was, however, no interaction of those factors. In Experiment 2, there was no effect of auditory cue, but there was a strong effect of event orientation with more patient-first structures produced when the action on the picture was right-to-left: X2(1) = 5.23, *p* = 0.022. Data of Experiment 3 is now collected and will be reported. Overall, these results as well as English language experiments suggest an existence of a hierarchy in effects of modality of primes on syntactic choice, with an interesting addition that Russian speakers tend to be more affected by event orientation than their English speaking counterparts.

**References**

- Gleitman, L. R., January, D., Nappa, R., & Trueswell, J. C. (2007). On give and take between event apprehension and utterance formulation. *Journal of Memory and Language*, 57(4), 544-569.
- Myachykov, A., & Tomlin, R. S. (2014). Attention and salience. In E. Dabrowska, & D. Divjak (Eds.). (2015). *Handbook of Cognitive Linguistics* (Vol. 39). Walter de Gruyter GmbH & Co KG.
- Spence, C. (2010). Crossmodal spatial attention. *Annals of the New York Academy of Sciences*, 1191(1), 182-200.
- Tomlin, R. S. (1995). Focal attention, voice, and word order: An experimental, cross-linguistic study. *Word Order in Discourse*, 517-554.

## POSTER SESSION: Visual search and scene perception

### Language mastery influences visual search strategies

Maria Rabeson, Irina Blinnikova, & Anna Izmalkova

Lomonosov Moscow State University, Russia

The study is an insight into the eye-movement patterns of language learners at different levels of mastery. Extended research into eye-movement patterns as connected to cognitive strategies in various tasks (Velichkovsky et al., 2005; Blinnikova et al., 2016) demonstrates that oculomotor correlates can indicate the characteristics of information processing (Rayner, 2009). Thus, the registration of eye-movement patterns while performing verbal search tasks reveals the peculiarities of cognitive strategies used by ESL (English as a Second Language) students at different levels of linguistic competence.

The hypothesis of the study is that a foreign language mastery level influences the cognitive strategies and oculomotor patterns applied by respondents in a verbal search task. This variation in strategy choices can be widely used as a marker of linguistic competence in a range of situations connected with education, especially with student assessment.

The experimental task included visual search for English words in letter matrices (15 x 15) with motor response (mouse clicks on the first and the last letter in the word found). The target stimuli were either horizontally or vertically oriented in equal proportion. Word frequency, length, and emotional valence were under control in the experiment. Respondents looked for words in 9 matrices, presented on the screen for 40 seconds each. Participants (45 people aged 18 to 33) were divided into three groups according to their English language mastery (as evaluated by self-report and Word Associates Test aimed at verbal competence in English). Subjects’ eye movements were recorded with an SMI RED 250 system. Mean comparison was applied to analyse the statistical data.

**Table 1.** Key parameters in verbal search

*Parameter*	*Group 1*	*Group 2*	*Group 3*	*Significance*
*Fixation Duration Average (ms)*	257.94	218.16	197.87	F (2;419) = 75.74 p<0.01
*Saccade Duration Average (ms)*	25.33	39.49	43.82	F (2;419) = 14.36 p<0.01
*Saccade Amplitude Average (°)*	3.22	4.99	6.35	F (2;419) = 18.06 p<0.01
*Words Found (#)*	1.11	2.00	2.43	F (2;419) = 33.36 p<0.01

The received data indicate that oculomotor patterns and visual search strategies differ significantly in the three groups of respondents. With all the participants reporting great cognitive strain to perform the task, A2 CEFR level students (group 1) demonstrate longer fixations and shorter saccades as compared to C1 students (group 3). The intermediary position of group 2 results (B1-B2 CEFR levels) support the hypothesis of gradual transition from focal to ambient strategies with the increase of language mastery. This study was sponsored by the RFBR research grant number 1636-00044.

**References**

- Blinnikova, I., Izmalkova, A., & Semenova, M. (2016). The factors of effectiveness and the organization of the search of elements within a graphic interface. *Information Content and Processing*, 3(2), 160-174.
- Velichkovsky, B. M., Joos, M., Helmert, J. R., & Pannasch, S. (2005). Two visual systems and their eye movements: Evidence from static and dynamic scene perception. In *Proceedings of the XXVII conference of the cognitive science society* (pp. 2283-2288).
- Rayner, K. (2009). The 35th Sir Frederick Bartlett Lecture: Eye movements and attention in reading, scene perception, and visual search. *Quarterly Journal of Experimental Psychology*, 62(8), 1457-1506.

### Measuring guided search parameters in ecological context with mobile eye tracking

Lykke Junker Andersen, Tea Hjermitslev Gjøderum, Peter Møller Nielsen, & Nik Kharlamov

Aalborg University, Denmark

Humans routinely look for specific objects among other objects, most of which are irrelevant to the task at hand. This process is known as visual search and is found in various settings such as assembling IKEA® furniture and looking for suspicious objects in airport security scanners.

A common theory of this process known as guided search (Wolfe, 2015) asserts that attentional deployment is “guided” by features of targets together with expectations about the likely locations of objects in a scene. One of the models of guided search where several targets are simultaneously searched among a large number of distractors (as in assembling LEGO® figures) is “hybrid foraging search” (Wolfe, Aizenman, Boettcher, & Cain, 2016), where foraging refers to the fact that many instances of targets held in memory could be present simultaneously. This model assumes an involvement of working memory (Drew, Boettcher, & Wolfe, 2016) and establishes a relationship between how many objects are held in memory (memory set size, MSS), how many distractors are present (visual set size, VSS), and reaction time (RT), such that RT is linearly related to VSS and the logarithm of MSS.

Experimental paradigms in the study of visual search usually involve stimuli presentation on a computer screen, with key-press- or mouse-click-based measurement of reaction time. The present study is an attempt to measure such reaction times in the ecological context of a real sensorimotor task – assembling simple LEGO® figures – where visual search theory predicts that search behaviour at the stage of looking for correct blocks will exhibit hybrid foraging characteristics.

A total of 12 participants wearing a Tobii® Pro Glasses 2 mobile eye tracker assembled LEGO® figures in a within-participant setup. Each participant assembled three figures of eight assembly steps each, with figures arranged in a Latin Square. For each step in assembly, the participants rotated on a swivelling chair between three different stations: instruction screen (presented on a laptop computer); search patch (tray with target and distractor blocks); and assembly desk (where the figure was built). The search patch was shuffled between steps. The participants’ gaze was tracked at 60 Hz sampling rate.

RT was extracted from gaze overlay videos, with trial start defined as first fixation on the LEGO® blocks in the search patch tray as the participant proceeded with assembly steps. In this presentation we discuss the applicability, validity, and robustness of four different RT measures with respect to establishing visual search model parameters: (1) RTF: From start of first fixation on previous target (p.t.) to start of first fixation on current target (c.t.); (2) RTS: From start of last fixation on p.t.to start of last fixation on c.t.; (3) RTT: From first touch of p.t. to first touch of c.t.; (4) RTFS: Average of RTF and RTS. We discuss model differences in terms of how these measures reflect different concepts of guided search termination.

**References**

- Drew, T., Boettcher, S. E., & Wolfe, J. M. (2016). Searching while loaded: Visual working memory does not interfere with hybrid search efficiency but hybrid search uses working memory capacity. *Psychonomic Bulletin and Review*, 23(1), 201-212.
- Wolfe, J. M. (2015). Visual search. In J. M. Fawcett, E. F. Risko, & A. Kingstone (Eds.), *The handbook of attention* (pp. 27-56). Cambridge, MA: The MIT Press.
- Wolfe, J. M., Aizenman, A. M., Boettcher, S. E., & Cain, M. S. (2016). Hybrid foraging search: Searching for multiple instances of multiple types of target. *Vision Research*, 119, 50-59. doi: 10.1016/j.visres.2015.12.006

### Task-dependency of eye movements in scene perception during quiet standing

Daniel Backhaus, Ralf Engbert, & Hans A. Trukenbrod

University of Potsdam, Germany

Scene viewing is used to study the dynamics of eye movement control; i.e., the overt allocation of attention. However, due to limitations of the scene viewing paradigm (e.g., missing task, use of a chin rest), the generalizability of experimental findings and their underlying theoretical assumptions to more natural tasks has been questioned (e.g., Tatler et al., 2011). Here, we investigate eye movements under less restricted conditions to explore the ecological validity of the scene viewing paradigm. Participants were standing in front of a projector screen and explored images under specific instructions: counting the number of people/animals in an image, or guessing the time/country when/where the image was taken. Eye movements were recorded using a mobile eye-tracking device; raw gaze data were transformed from head-centred into image-centred coordinates before saccade detection. Epochs with low quality gaze trajectories were removed from further analyses. We evaluated our pre-processing routines to ensure high quality for the detection of saccades and fixations (cf. main sequence, Figure 1a). In general, eye movements during standing in front of a projector resembled scene viewing under more controlled conditions. In addition, task manipulations led to reliable differences in saccade amplitudes (Figure 1b) and fixation durations (Figure 1c) between task conditions. Our results lend support to the view that findings from highly controlled laboratory experiments can be reproduced under more relaxed and thus more natural setups.

**Figure. 1:**
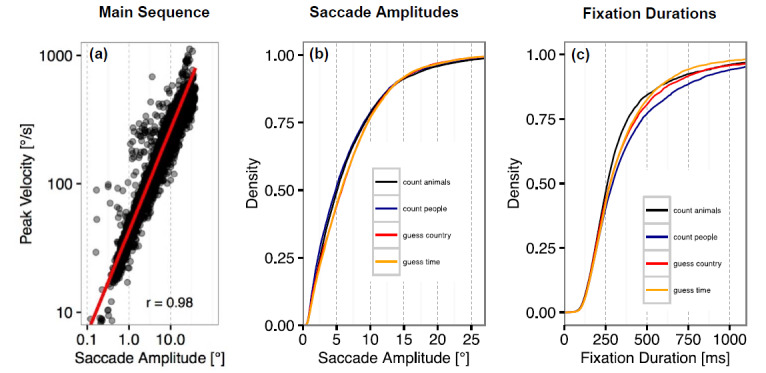
Results of the scene viewing experiment: (a) Main Sequence; (b) Saccade Amplitudes; (c) Fixation Durations.

**References**

- Tatler, B. W., Hayhoe, M. M., Land, M. F., & Ballard, D. H. (2011). Eye guidance in natural vision: Reinterpreting salience. *Journal of Vision*, 11(5):5, 1-23. doi: 10.1167/11.5.5

### Visual search strategies in expert vs. non-expert trail runners

Ricardo Gomes^1,2^, Manuel J Coelho-e-Silva^2,3^, Rui Mendes^1,3^, & Gonçalo Dias^1,3^

^1^ Polytechnic Institute of Coimbra, ESEC, ASSERT, Portugal

^2^ FCDEF, University of Coimbra, Portugal

^3^ CIDAF, University of Coimbra, Portugal

The ability to detect predictive visual information is central to elite performance. The comparison between the number of fixations and fixation times in experts and non-experts has produced two opposing views. On the one hand, Mann, Williams, Ward and Janelle (2007), found that expert athletes tend to have fewer and longer fixations. On the other hand, the opposite has also been observed (e.g., Manzanares, Menayo, & Segado, 2017). The aim of this study was to analyse the differences in gaze behaviour between expert and non-expert trail running athletes when running on uneven terrain under two conditions: rested and fatigued. A total of 15 trail running athletes – eight experts (40.75 ± 3.67) and seven non-experts (37.00 ± 5.24) – ran on a trail running test track using head-mounted eye-tracking glasses. Fixations on Areas of Interest and Fixation Times were compared between experts and non-experts, and within these groups, for the two conditions: rested and fatigued. Experts had significantly fewer total fixations than non-expert athletes for the rested condition (*t* = 3.010, *p* < 0.05, *d* = 1.56). No significant differences between groups were found for the fixation time. Considering the results, we conclude that expert runners need to look less to the terrain to draw the necessary information to cope with it. Additionally, fatigue did not affect visual behaviour in any group. Further studies should focus on assessing whether this visual behaviour affects the kinematics of movement.

Key words: gaze behaviour, eye movements, trail running, visual search strategies, perception-action

**References**

- Mann, D. T., Williams, A. M., Ward, P., & Janelle, C. M. (2007). Perceptual-cognitive expertise in sport: A meta-analysis. *Journal of Sport & Exercise Psychology*, 29(4), 457-478. doi: 10.1123/jsep.29.4.457
- Manzanares, A., Menayo, R., & Segado, F. (2017). Visual search strategy during regatta starts in a sailing simulation. *Motor Control*, 21(4), 413-424. doi: 10.1123/mc.2015-0092

## POSTER SESSION: Consumer behaviour and logical reasoning

### Exploring the role of wine packaging aesthetics: A cross-cultural study using visual attention as indirect measure of preference

Andrea Ciceri¹, Vincenzo Russo¹, & Jesper Clement²

¹ IULM University, Milan, Italy

² Copenhagen Business School, Denmark

**Abstract:**

Wine consumers rely mainly on the packaging and label to infer the quality of wine and make a choice of a bottle. The results from a study conducted in Denmark and Italy combining traditional questionnaire and a new method based on visual data, reported a comparison in terms of preferences about different wine packaging elements. The decision-making process has been explored too.

**Research question:**

The main objective of the research was to compare the decision-making process of different consumers (Danish and Italian) with respect to wine packaging, classified in terms of modern and traditional appearance. The main hypotheses were two: (1) Danish consumers prefer modern labels. Italians prefer more the traditional labels; and (2) Danish consumers have a higher level of decision-making difficulty in comparison to the Italians.

**Method:**

From a methodological point of view, various methods have been used to determine wine consumers’ behaviour and buying habits. A major unresolved research question is how wine packaging preference and importance can be reliably and validly measured without the risk of participants hiding their answers. In order to take this problem into account, a new methodology has been adopted, using the visual data as an implicit and objective way to infer the participants’ preferences about different wine packaging elements. Moreover, we used a skin conductance signal to detect the objective difficulty in the decision-making process. A questionnaire was used to collect information about other aspects of the decision-making process. A total of 120 participants took part in the research.

**Results and discussion:**

Results confirmed that traditional labels are preferred more by both Italians and Danish. Eye- tracking data and self-report data converge in this direction. Probably both samples have preferred wine with traditional labels in order to reduce the perceived risk.

On the other hand, significant differences were verified with regard to decision-making difficulties. Danish consumers experienced a higher level of decision-making difficulty. These results can be explained by taking into consideration the different consumption habits that Danish and Italian consumers have. Danish consumers, having less confidence with wine products, also had a greater level of difficulty in completing this simple decision-making task.

Key words: visual preference detection, eye tracker, wine packaging

### What do your eyes reveal about counterfactual conditionals? An easy context for negation

Isabel Orenes¹, Juan Antonio García-Madruga¹, Orlando Espino², & Ruth Byrne³

¹ UNED University, Madrid, Spain

² University of La Laguna, Tenerife, Spain

³ Trinity College, Dublin, Republic of Ireland

We report an experiment to examine how people understand affirmative and negative counterfactual conditionals. Participants listened to short stories: for example, “Miguel went to a flower shop and did not know whether to buy roses or carnations.” They heard a critical sentence such as, “If he had arrived early, he would have bought roses”. We compared affirmative causal assertions, e.g., “Because he arrived early, he bought roses”, negative causal assertions, “Because he did not arrive early, he did not buy roses”, to affirmative counterfactual conditionals, “If he had arrived early, he would have bought roses”, and negative counterfactual conditionals, “If he had not arrived early, he would have not bought roses”. Participants listened to the stories while looking at four printed words on a computer screen: e.g., “roses”, “no roses”, “carnations”, “no carnations”. We used eye-tracking methods to examine where their eyes looked while they heard the target sentences. The results showed that participants looked at the target word “roses” in the affirmative causal conditionals faster than when they looked at the alternative “carnations” in the negative causal. On the other hand, they looked at “roses” in the negative counterfactual conditionals faster than when they looked at first “roses” and then “carnations” in the affirmative counterfactual. We discuss the implications of the results for the dual meaning account of counterfactuals.

## Footnotes

www.visionbuilder.no, last visited 28-04-2018.

www.markersoftware.com, last visited 29-06-2018.

CompAsS (Computer-Assisted Subtitling) is a Horizon 2020 project funded by the EU under reference CNECT 2017/3135124-4 in cooperation with ZDF Digital Medienproduktion GmbH.

F1 score balances precision and recall and is a useful evaluation metric when class distributions are skewed.

